# Comprehensive Genomic Characterization Between Urothelial Carcinoma Subtypes/Divergent Differentiation (S/DD) and Pure Urothelial Carcinoma Using a Large‐Scale Japanese Genomic Panel Dataset

**DOI:** 10.1111/iju.70538

**Published:** 2026-06-08

**Authors:** Go Kobayashi, Yohei Sekino, Shunsuke Miyamoto, Kohei Kobatake, Hiroyuki Kitano, Keisuke Goto, Akihiro Goriki, Keisuke Hieda, Testutaro Hayashi, Kazuhiro Sentani, Nobuyuki Hinata

**Affiliations:** ^1^ Department of Pharmacy, Faculty of Pharmacy Yasuda Women's University Hiroshima Japan; ^2^ Department of Urology, Graduate School of Biomedical and Health Sciences Hiroshima University Hiroshima Japan; ^3^ Department of Molecular Pathology, Graduate School of Biomedical and Health Sciences Hiroshima University Hiroshima Japan

**Keywords:** C‐CAT, center for cancer genomics and advanced therapeutics, gene mutation, oncoplot, subtypes/divergent differentiation, urothelial carcinoma

## Abstract

**Objectives:**

Urothelial carcinoma (UC) is a common malignancy; however, UC subtypes/divergent differentiation (S/DD) accounts for only 10%–20% of overall cases. S/DD's aggressive biological behavior significantly affects its prognosis and therapeutic decision‐making; thus, elucidating its genomic landscape within UC is important. This retrospective observational study aimed to evaluate and compare the molecular characteristics of S/DD and pure urothelial carcinoma (PUC).

**Methods:**

Comprehensive cancer genomic profiling data were obtained from the Center for Cancer Genomics and Advanced Therapeutics (C‐CAT) and the MSK2022 dataset. The association between S/DD and clinicopathological features was evaluated in an independent Hiroshima University cohort.

**Results:**

Across both C‐CAT and MSK2022 datasets, *TP53* and *RB1* mutations were more prevalent in S/DD, whereas *KDM6A*, *ARID1A*, and *FGFR3* mutations were more common in PUC. High‐frequency copy number alterations included *MYC* and *RB1* in S/DD, and *CDKN2A*, *CDKN2B*, *CCND1*, *FGF19*, *FGF4*, and *FGF3* in PUC. In the Hiroshima University cohort, S/DD was associated with adverse clinicopathological features and a poor prognosis. Immunohistochemistry demonstrated a positive correlation of S/DD with PD‐L1, EGFR, and p53 expression in upper tract UC tissues, and an inverse correlation with FGFR3, GATA3, Nectin4, and TROP2 expression.

**Conclusions:**

Using a large‐scale Japanese genomic panel dataset, we characterized the molecular alterations associated with S/DD. S/DD frequently exhibits low Nectin‐4 expression and basal‐like molecular features, which may have implications for treatment selection and inform future therapeutic strategies.

## 1 Introduction

Urothelial carcinoma (UC) is a common malignancy that arises in the urinary bladder (BLCA) and upper urinary tract (UTUC). Histologically, UC comprises pure urothelial carcinoma (PUC) and tumors exhibiting histological subtypes/divergent differentiation (S/DD). S/DD includes squamous, glandular, micropapillary, plasmacytoid, sarcomatoid, and small cell variants, among others [1]. Although relatively uncommon, S/DD is more aggressive and associated with poorer outcomes than PUC [1]. Therefore, elucidating the underlying molecular mechanisms of S/DD and identifying novel therapeutic targets are of great clinical importance.

Molecular classifications such as mutational and consensus subtypes have been proposed for UC [2, 3]. Although S/DDs, such as squamous differentiation, are generally categorized within the basal molecular subtype, several S/DDs are difficult to assign to a specific molecular subtype due to their diverse histological features [1]. Several studies have explored the relationship between distinct S/DD and gene mutations. For instance, Chu et al. demonstrated that *ERBB2*, *FGFR3*, and *PTEN* alterations were more frequent in micropapillary, nested/squamous, and sarcomatoid UC, respectively, whereas tumor mutational burden (TMB) was highest in plasmacytoid, neuroendocrine, and micropapillary tumors [4]. Other studies showed that UC with squamous differentiation is frequently associated with *TP53* mutations and rarely with *FGFR3* mutations, and sarcomatoid and small cell variants have been reported to frequently harbor *TP53* and *RB1* mutations [2, 5, 6, 7]. These findings highlight the heterogeneous genomic landscape of S/DD.

Recent pathological consensus recommendations emphasize that divergent differentiation and distinct histological subtypes are biologically distinct entities and should not be grouped indiscriminately [8]. Nevertheless, current treatment strategies for UC are not stratified according to histological subtype. This may partly reflect the limited molecular characterization of each variant, the small number of cases, and low reproducibility of histological classification. Given the practical difficulty of classifying all S/DDs into detailed histological subtypes and analyzing their genomic features individually, it is crucial to recognize the overall mutational trends of S/DDs. Notably, a recent single‐cell RNA sequencing study stratified UC into four PUC subtypes and eleven histologically distinct S/DD categories and demonstrated that, despite marked histological heterogeneity, S/DDs share common molecular features, including preservation of a CA125‐positive tumor cell subpopulation [9]. These findings suggest that integrated analyses of S/DDs may still yield meaningful insights. Accordingly, while subtype‐specific analyses remain essential, identifying genomic features shared among S/DDs may provide clinically relevant insights for therapeutic decision‐making and biomarker development. However, large‐scale real‐world genomic analyses integrating histological classification with molecular profiling, particularly distinguishing histological subtypes from divergent differentiation, remain limited.

To clarify the nationwide genetic landscape of S/DD, we conducted a retrospective observational study using comprehensive cancer genomic profiling data collected by the Center for Cancer Genomics and Advanced Therapeutics (C‐CAT) [10]. We additionally analyzed data from the large‐scale Memorial Sloan Kettering Cancer Center (MSK) UC cohort [11] to investigate somatic alteration profiles characteristic of S/DD.

## 2 Materials and Methods

### 2.1 The C‐CAT and MSK Datasets

We retrospectively analyzed genomic data from the C‐CAT database [10] and the MSK2022 dataset [11]. Detailed information on patient selection, sequencing platforms, and histological classification is provided in the Supplementary Method 1. In the C‐CAT cohort, 1292 UC cases were classified as PUC (*n* = 1073) or S/DD (*n* = 219). S/DD cases were further categorized into divergent differentiation (*n* = 108) and distinct histological subtypes (*n* = 111). In the MSK2022 cohort, 1656 UC samples included 1621 PUC and 35 S/DD cases, subdivided into divergent differentiation (*n* = 14) and distinct histological subtypes (*n* = 21) (Tables 1 and 2).

**TABLE 1:** Distribution of urothelial carcinoma subtypes/divergent differentiation (S/DD) in this study.

C‐CAT database	MSK2022 database	Hiroshima cohort
Squamous differentiation (*n* = 60)	Squamous differentiation (*n* = 6)	Squamous differentiation (*n* = 20)
Glandular differentiation (*n* = 47)	Glandular differentiation (*n* = 8)	Glandular differentiation (*n* = 2)
Neuroendocrine carcinoma (*n* = 71)	Neuroendocrine carcinoma (*n* = 9)	Neuroendocrine carcinoma (*n* = 1)
Plasmacytoid/Signet Ring Cell (*n* = 18)	Plasmacytoid/Signet Ring Cell (*n* = 5)	Sarcomatoid (*n* = 3)
Micropapillary (*n* = 9)	Sarcomatoid (*n* = 1)	Micropapillary (*n* = 2)
Sarcomatoid (*n* = 5)	Poorly differentiated (*n* = 2)	Trophoblastic differentiation (*n* = 1)
Clear cell (*n* = 5)	Undifferentiated (*n* = 4)	
Poorly differentiated (*n* = 3)		
Trophoblastic differentiation (*n* = 1)		
219 (17%) of 1292 cases	35 (2%) of 1656 cases	29 (12%) of 246 cases

**TABLE 2:** Distribution of divergent differentiation and histological subtypes.

C‐CAT database	MSK2022 database
Divergent differentiation (*n* = 108)	Divergent differentiation (*n* = 14)
Squamous differentiation (*n* = 60)	Squamous differentiation (*n* = 6)
Glandular differentiation (*n* = 47)	Glandular differentiation (*n* = 8)
Trophoblastic differentiation (*n* = 1)	
Histological subtypes (*n* = 111)	Histological subtypes (*n* = 21)
Neuroendocrine carcinoma (*n* = 71)	Neuroendocrine carcinoma (*n* = 9)
Plasmacytoid/Signet Ring Cell (*n* = 18)	Plasmacytoid/Signet Ring Cell (*n* = 5)
Micropapillary (*n* = 9)	Sarcomatoid (*n* = 1)
Sarcomatoid (*n* = 5)	Poorly differentiated (*n* = 2)
Clear cell (*n* = 5)	Undifferentiated (*n* = 4)
Poorly differentiated (*n* = 3)	

### 2.2 Analysis of UTUC and BLCA in the Hiroshima University Cohort

The medical records of patients who underwent radical nephroureterectomy for unilateral UTUC (*n* = 153) or radical cystectomy (RC) for BLCA (*n* = 93) at Hiroshima University Hospital are detailed. The details of patient information and the immunohistochemical analysis of various molecules have been described in Supplementary Method 1 and previous studies [12, 13, 14, 15, 16]. S/DD was indicated in 12 of 153 UTUC cases and 17 of 93 BLCA cases. The proportion of S/DD differed between the UTUC and BLCA cohorts, which may partly reflect differences in clinical case composition. The UTUC cohort included a substantial number of non–muscle‐invasive tumors, whereas the BLCA cohort primarily consisted of patients who underwent radical cystectomy and therefore included mainly muscle‐invasive disease. Details of S/DD subtypes across all datasets are summarized in Table 1.

In this study, squamous cell carcinomas were classified by squamous differentiation, adenocarcinomas by glandular differentiation, and small cell carcinomas by neuroendocrine differentiation.

### 2.3 Statistical Analysis

All statistical analyses were performed using SPSS (SPSS Inc., Chicago, IL, USA). Clinical and genomic variables were compared between two groups using the Mann–Whitney U test, and *p* values < 0.05 were considered statistically significant. Oncoplots were generated using Python (ver. 3.0) within the Jupyter Notebook environment (ver. 6.3.0). The scripts used are provided in the Supplementary Method 2. Correlations between clinicopathological parameters and S/DD in the Hiroshima University cohort were analyzed using Fisher's exact test. Kaplan–Meier survival curves were constructed, and differences in survival were evaluated using the log‐rank test.

## 3 Results

### 3.1 Comparison of Clinical and Genomic Variables Between S/DD and PUC

**FIGURE 1:**
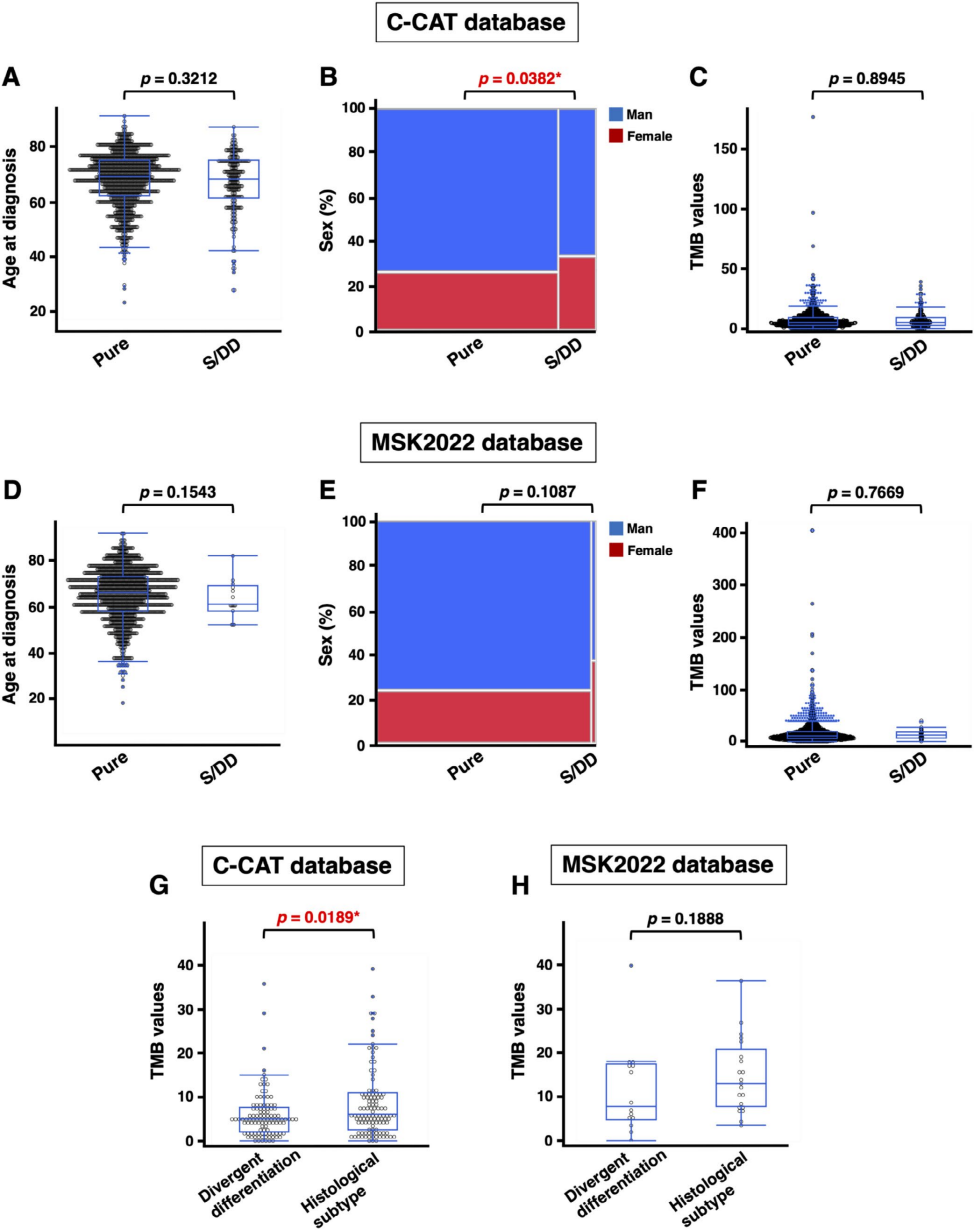
Clinical data and tumor mutation burden (TMB) of urothelial carcinoma subtypes/divergent differentiation (S/DD) and pure urothelial carcinoma (PUC). Comparison of (A) age, (B) sex and (C) TMB score between S/DD and PUC in the C‐CAT dataset. Comparison of (D) age, (E) sex and (F) TMB score between S/DD and PUC in the MSK2022 dataset. Comparison of TMB score between histological subtypes and divergent differentiation in the (G) C‐CAT and (H) MSK2022 dataset. *p* values were calculated using Mann–Whitney U test or Fisher's exact test, with statistically significant differences highlighted in red. Asterisks indicate statistical significance (*p < 0.05).

In the C‐CAT dataset [10], patient age did not differ significantly between PUC and S/DD (Figure 1A). The frequency of S/DD was significantly higher in female patients (Figure 1B). There was no significant difference in TMB between the two groups (Figure 1C). Similar trends were observed in the MSK2022 dataset (Figure 1D–F). Notably, subgroup analyses revealed that TMB was significantly higher in distinct histological subtypes than in tumors with divergent differentiation in the C‐CAT dataset (Figure 1G). While this difference did not reach statistical significance in the MSK2022 dataset due to the limited number of cases, a similar trend was observed (Figure 1H). Additionally, the analysis of the C‐CAT dataset revealed that the numbers of somatic mutations and gene rearrangements were significantly higher in PUC than in S/DD (Figure S1).

### 3.2 Oncoplot Analysis in the C‐CAT Dataset

**FIGURE 2:**
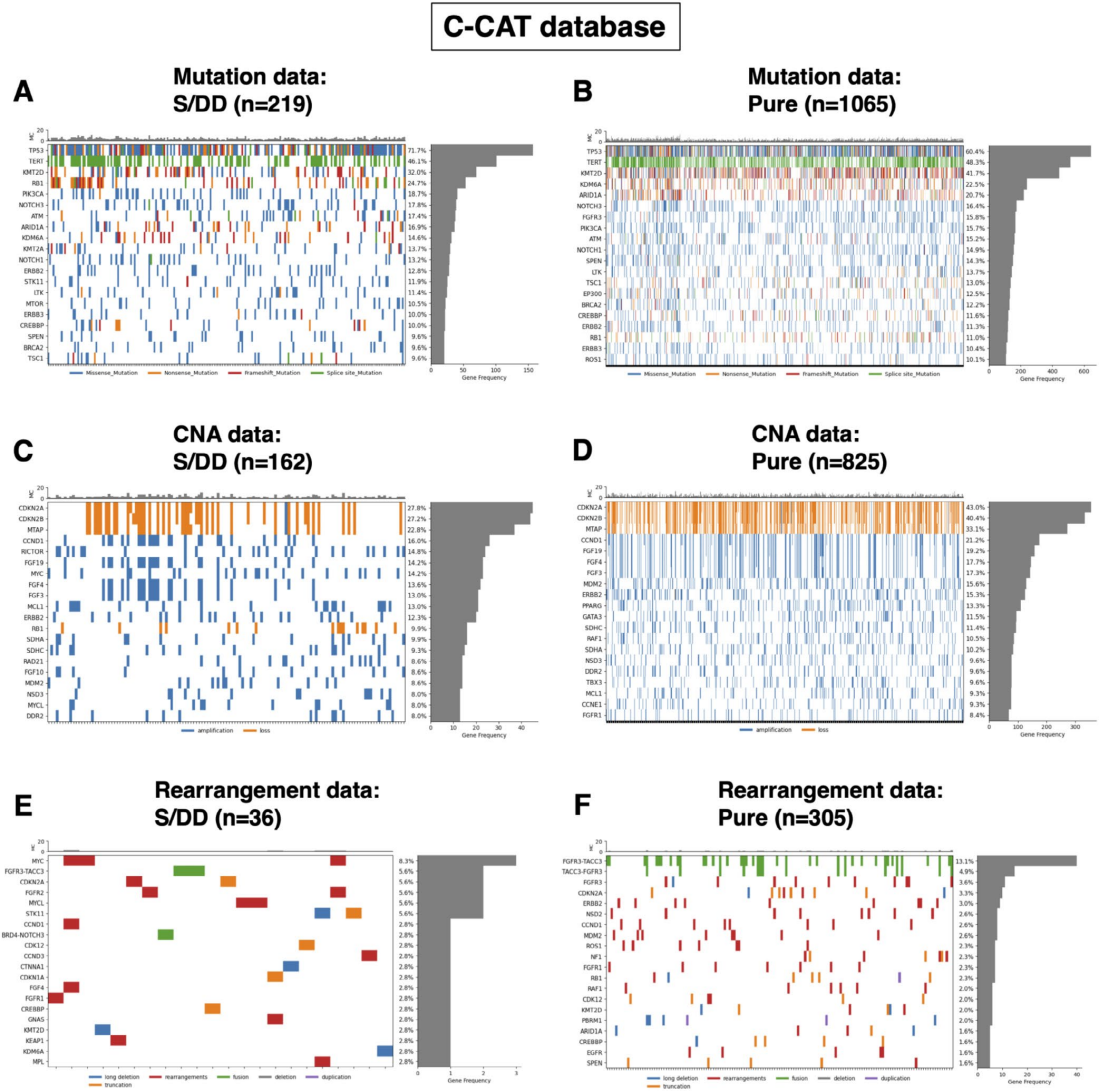
Genomic profiles of urothelial carcinoma subtypes/divergent differentiation (S/DD) and pure urothelial carcinoma (PUC) in the C‐CAT dataset. Oncoplots showing somatic mutations in frequently altered genes in (A) S/DD (*n* = 219) and (B) PUC (*n* = 1065). Oncoplots showing copy number alterations (CNAs) in (C) S/DD (*n* = 162) and (D) PUC (*n* = 825). Oncoplots showing gene rearrangements in (E) S/DD (*n* = 36) and (F) PUC (*n* = 305).

We next compared the somatic mutation profiles between the two groups using an oncoplot. Notably, mutations of *TP53*, *RB1*, and *KMT2A* were more prevalent in S/DD, while mutations of *KMT2D*, *KDM6A*, *ARID1A*, and *FGFR3* were more frequent in PUC (Figure 2A,B).

In the CNA dataset, amplifications or deletions in *RICTOR*, *MYC*, *RB1*, and *RAD21* were predominant in S/DD. Conversely, PUC showed a higher frequency of CNAs in various genes, including *CDKN2A*, *CDKN2B*, *MTAP*, *CCND1*, *FGF19*, *FGF4*, *FGF3*, *MDM2*, and *PPARG* (Figure 2C,D).

Although the number of rearrangement events was limited, rearrangements of *MYC* and *MYCL* were more frequent in S/DD. In contrast, FGFR3‐related fusions, including *FGFR3–TACC3* and *FGFR3*, were observed exclusively in PUC (Figure 2E,F).

### 3.3 Oncoplot Analysis in the MSK2022 Dataset

**FIGURE 3:**
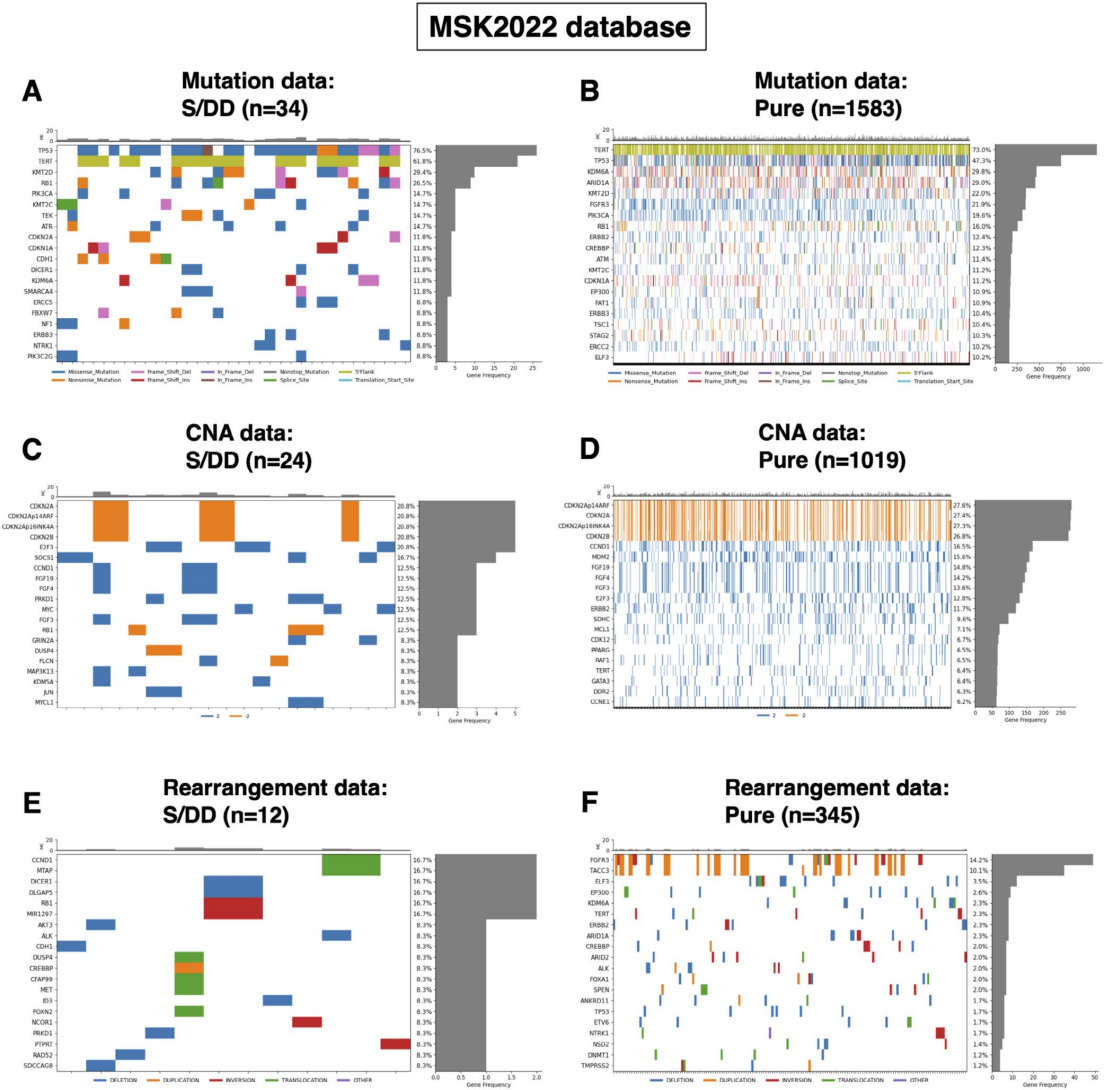
Genomic profiles of urothelial carcinoma subtypes/divergent differentiation (S/DD) and pure urothelial carcinoma (PUC) in the MSK2022 dataset. Oncoplots showing somatic mutations in frequently altered genes in (A) S/DD (*n* = 34) and (B) PUC (*n* = 1583). Oncoplots showing copy number alterations (CNAs) in (C) S/DD (*n* = 24) and (D) PUC (*n* = 1019). Oncoplots showing gene rearrangements in (E) S/DD (*n* = 12) and (F) PUC (*n* = 345).

We additionally analyzed the mutational landscape of S/DD using the MSK2022 dataset [11]. The oncoplot analysis of somatic mutation profiles revealed that mutations in *TP53*, *RB1*, and *KMT2D* were more prevalent in S/DD, whereas mutations in *TERT*, *KDM6A*, *ARID1A*, and *FGFR3* were more frequent in PUC (Figure 3A,B).

In the CNA dataset, CNAs involving *E2F3*, *RB1*, *MYC*, and *PRKD1* were more frequent in S/DD. Conversely, CNAs affecting *CDKN2Ap14ARF*, *CDKN2A*, *CDKN2Ap16INK4A*, *CDKN2B*, *MDM2*, *ERBB2*, and *SDHC* were more frequent in PUC (Figure 3C,D).

The structural variation oncoplot revealed that alterations in *RB1*, *MIR1297*, *DICER1*, *MTAP*, and *CCND1* were more prevalent in the S/DD, while alterations in *FGFR3* and *TACC3* were more frequent in the PUC (Figure 3E,F).

### 3.4 Oncoplot Analysis of S/DD Subgroups

**FIGURE 4:**
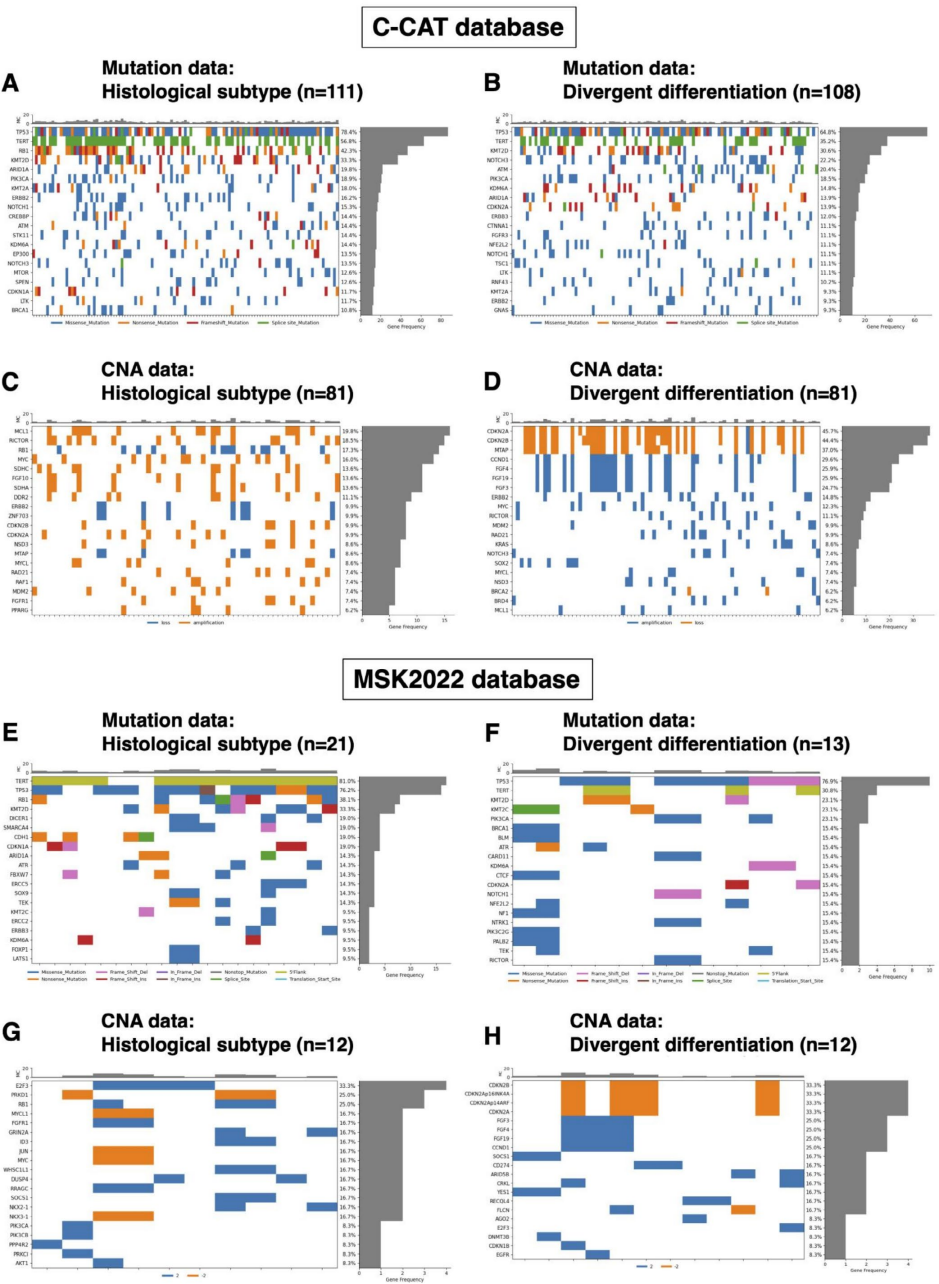
Genomic profiles of urothelial carcinoma subgroups defined by histological subtypes and divergent differentiation. Oncoplots showing somatic mutations in frequently altered genes in (A) histological subtypes (*n* = 111) and (B) divergent differentiation (*n* = 108) in the C‐CAT dataset. Oncoplots showing copy number alterations (CNAs) in (C) histological subtypes (*n* = 81) and (D) divergent differentiation (*n* = 81) in the C‐CAT dataset. Oncoplots showing somatic mutations in frequently altered genes in (E) histological subtypes (*n* = 21) and (F) divergent differentiation (*n* = 13) in the MSK2022 dataset. Oncoplots showing CNAs in (G) histological subtypes (*n* = 12) and (H) divergent differentiation (*n* = 12) in the MSK2022 dataset.

We attempted to further subgroup analyses of S/DD according to histological subtypes and divergent differentiation. In the C‐CAT dataset, mutations in *TP53*, *TERT*, *RB1*, *ARID1A*, and *KMT2A* were more prevalent in tumors with histological subtypes. In contrast, mutations in *NOTCH3*, *ATM*, *CDKN2A*, and *NFE2L2* were more frequently observed in tumors with divergent differentiation (Figure 4A,B).

Analysis of CNAs demonstrated that alterations involving *MCL1*, *RICTOR*, *RB1*, *SDHC*, *SDHA*, and *FGF10* were more common in histological subtypes. Conversely, CNAs affecting *CDKN2A*, *CDKN2B*, *MTAP*, *CCND1*, *FGF19*, *FGF4*, and *FGF3* were more frequently detected in divergent differentiation (Figure 4C,D).

Although the number of cases was limited, we also evaluated the analysis of S/DD subgroups using the MSK2022 dataset. Mutations in *TERT* and *RB1* were more prevalent in tumors with histological subtypes, whereas mutations in *KMT2C* and *PIK3CA* were more frequently observed in divergent differentiation (Figure 4E,F).

CNA analysis of the MSK2022 dataset showed that alterations involving *E2F3*, *RB1*, and *PRKD1* were more frequent in histological subtypes. Conversely, CNAs affecting *CDKN2A*, *CDKN2B*, *CDKN2Ap14ARF*, *CDKN2Ap16INK4A*, *CCND1*, *FGF19*, *FGF4*, and *FGF3* were more frequent in divergent differentiation (Figure 4G,H). Detailed results of the oncoplot analyses shown in Figures 2, 3, 4 are provided in Tables S1–, S4.

### 3.5 Subgroup Analyses Separating UTUC and BLCA Cases

UTUC exhibited a higher frequency of KMT2D alterations and a lower frequency of TERT alterations than BLCA (Figure S2A–D). TP53 and RB1 alterations remained more frequent in S/DD in both UTUC and BLCA (Figure S2A–D). Regarding CNAs, BLCA showed a higher frequency of ERBB2 alterations than UTUC (Figure S2E–H). Although S/DD and PUC in UTUC showed broadly similar profiles, S/DD tended to show higher MYC alterations and lower GATA3 alterations (Figure S2G,H).

In comparisons between histological subtypes and divergent differentiation, both UTUC and BLCA demonstrated higher frequencies of RB1 and TERT alterations in histological subtypes (Figure S3A–D). In UTUC, histological subtypes also showed a higher frequency of KMT2D alterations (Figure S3C,D). Although copy number patterns among histological subtypes were somewhat heterogeneous, divergent differentiation showed relatively similar profiles in both UTUC and BLCA (Figure S3E–H).

### 3.6 Subtype‐Stratified Analyses

The additional oncoplot analysis using the C‐CAT dataset was conducted in each S/DD subtype. It revealed distinct subtype‐specific genomic patterns (Figure S4A–F, Table S5). For example, glandular differentiation demonstrated increased ATM alterations together with reduced TERT and KMT2D alterations, while squamous differentiation exhibited a more moderate but distinct genomic profile (Figure S4A,B). Neuroendocrine carcinoma was characterized by frequent TP53 and RB1 alterations, whereas micropapillary variants showed enrichment of ERBB2 alterations (Figure S4C,D). Plasmacytoid/signet ring cell tumors showed alterations involving CDH1‐related and cell‐cycle pathways (Figure S4E). We also evaluated CNAs, which further supported biological heterogeneity among S/DD (Figure S5A–F, Table S5).

### 3.7 Relationship Between S/DD and Clinicopathological Features

In the MSK2022 dataset, S/DD was significantly associated with a poor prognosis (Figure S6A). In the Hiroshima University cohort, S/DD was significantly associated with nodular/flat morphology in both UTUC and BLCA (Table 3). Additionally, S/DD was significantly associated with decreased CSS and PFS in UTUC, as well as shorter CSS in BLCA (Figure S6B–D). We further investigated the association between S/DD and various cancer‐related molecules in the UTUC dataset using an immunohistochemical analysis. S/DD was positively associated with the expression of PD‐L1, EGFR, and p53 in UTUC tissues, while it was inversely associated with the expression of FGFR3, GATA3, Nectin4, and TROP2 (Figure S7, Table 4).

**TABLE 3:** Relationship between urothelial carcinoma subtypes/divergent differentiation (S/DD) and clinicopathological characteristics in 153 cases of upper tract urothelial carcinoma (UTUC) and 93 cases of bladder urothelial carcinoma (BLCA) in Hiroshima cohort.

Factors in UTUC cohort	S/DD	Pure	*p*
Age			
< 73 years (*n* = 77)	5 (6%)	72 (94%)	0.5638
≥ 73 years (*n* = 76)	7 (9%)	69 (91%)	
Sex			
Female (*n* = 34)	4 (12%)	30 (88%)	0.4672
Man (*n* = 119)	8 (7%)	111 (93%)	
Morphology			
Papillary (*n* = 87)	1 (1%)	86 (99%)	**0.0004**
Nodular/Flat (*n* = 66)	11 (17%)	55 (83%)	
Pathological T stage			
pTa/is/1 (*n* = 80)	0 (0%)	80 (100%)	**< 0.0001**
pT2/3/4 (*n* = 73)	12 (16%)	61 (84%)	

Factors in BLCA cohort	S/DD	Pure	*p*
Age			
< 73 years (*n* = 47)	6 (13%)	41 (87%)	0.1891
≥ 73 years (*n* = 46)	11 (24%)	35 (76%)	
Sex			
Female (*n* = 25)	7 (28%)	18 (72%)	0.2239
Man (*n* = 68)	10 (15%)	58 (85%)	
Morphology			
Papillary (*n* = 28)	0 (0%)	28 (100%)	**0.0023**
Nodular/Flat (*n* = 65)	17 (26%)	48 (74%)	
Pathological T stage			
pT0/1/2 (*n* = 47)	6 (13%)	41 (87%)	0.1891
pT/3/4 (*n* = 46)	11 (24%)	35 (76%)	

*Note:* *p* values were calculated with Fisher's exact test. Bold values show the statistical significance at the *p* < 0.05 level.

**TABLE 4:** Relationship between urothelial carcinoma subtypes/divergent differentiation (S/DD) and various cancer‐related molecules in 153 cases of upper tract urothelial carcinoma.

	S/DD	Pure	*p*
Ki‐67			
Positive (> 20%)	5 (12%)	38 (88%)	0.3193
Negative (≤ 20%)	7 (6%)	103 (94%)	
PD‐L1 in TCs			
Positive	6 (29%)	15 (71%)	**0.0018**
Negative	6 (5%)	126 (95%)	
PD‐L1 in TILs			
Positive	6 (13%)	41 (87%)	0.1895
Negative	6 (6%)	100 (94%)	
CD8 in TILs			
Positive	4 (8%)	46 (92%)	1.0000
Negative	8 (8%)	95 (92%)	
HER2			
Positive	4 (14%)	24 (86%)	0.2339
Negative	8 (6%)	117 (94%)	
EGFR			
Positive	6 (32%)	13 (68%)	**0.0010**
Negative	6 (4%)	128 (96%)	
FGFR3			
Positive	0 (0%)	47 (100%)	**0.0184**
Negative	12 (11%)	94 (89%)	
p53			
Positive	7 (15%)	39 (85%)	**0.0442**
Negative	5 (5%)	102 (95%)	
GATA3			
Positive	5 (4%)	130 (96%)	**< 0.0001**
Negative	7 (39%)	11 (61%)	
UPK3			
Positive	1 (2%)	50 (98%)	0.0622
Negative	11 (11%)	91 (89%)	
CK 5/6			
Positive	5 (16%)	26 (84%)	0.0679
Negative	7 (6%)	115 (94%)	
Nectin‐4			
Positive	6 (5%)	117 (95%)	**0.0180**
Negative	5 (21%)	19 (79%)	
TROP2			
Positive	2 (3%)	70 (97%)	**0.0499**
Negative	6 (14%)	36 (86%)	

*Note:* *p* values were calculated with Fisher's exact test. Bold values show the statistical significance at the *P* < 0.05 level.

Abbreviations: EGFR, epidermal growth factor receptor; FGFR3, fibroblast growth factor receptor 3; HER2, human epidermal growth factor receptor type 2; PD‐L1, programmed death ligand 1; TCs, tumor cells; TILs, tumor‐infiltrating lymphocytes.

## 4 Discussion

The C‐CAT, established in 2019 under Japan's national health insurance system, functions as the national data center for cancer genomic medicine by integrating clinical information with comprehensive genomic profiling data from patients who have provided written informed consent [10]. Leveraging this nationwide resource, we performed a comprehensive real‐world genomic analysis integrating histopathological classification with molecular profiling in S/DD and PUC. We found that the frequency of S/DD was significantly higher in females than in males. Females tend to be diagnosed at more advanced disease stages in UC [17], and because S/DD is associated with higher pathological stage [18], this stage distribution may partly explain the observed sex difference. However, biological or hormonal factors may also contribute, warranting further investigation. Additional stratified analyses confirmed established molecular differences between UTUC and BLCA, with BLCA showing more frequent TERT and ERBB2 alterations, whereas KMT2D alterations were enriched in UTUC. Our results also showed no significant difference in TMB values between S/DD and PUC. However, subgroup analysis showed that TMB was significantly higher in tumors with distinct histological subtypes than in those with divergent differentiation. Since higher TMB is generally associated with increased neoantigen load and improved immune recognition, it has been proposed as a predictive biomarker for response to immunotherapy [19]. Previous studies have reported elevated TMB in small cell variant UC [7], whereas no significant difference in TMB has been found between the micropapillary subtype and PUC [20]. In contrast, a recent genomic landscape analysis has shown that TMB was highest in plasmacytoid, neuroendocrine, and micropapillary tumors. Thus, further investigations are necessary to elucidate the relationship between S/DD and TMB.

In this study, TP53, RB1, and MYC mutations were more frequent in S/DD, while *FGFR3* mutations were less common. The findings for TP53 and FGFR3 were consistent with the immunohistochemical results from the Hiroshima University cohort. MYC amplification and overexpression have been associated with tumor progression and poor prognosis in UC [21], and MYC alterations are also implicated in treatment resistance in several cancers [22]. Moreover, the KLF16–MYC pathway has been reported to regulate tumor proliferation and sensitivity to cisplatin/gemcitabine in UC, suggesting the potential therapeutic relevance of targeting MYC‐related pathways [23]. These findings indicate that MYC dysregulation may represent a potential therapeutic target in S/DD.

Our subgroup analysis further demonstrated the biological heterogeneity within S/DD. When S/DD was stratified into tumors with distinct histological subtypes and those with divergent differentiation, different mutational landscapes became apparent. Histological subtypes showed higher frequencies of TP53, RB1, and TERT mutations, suggesting a more proliferative and genomically unstable phenotype. In contrast, tumors with divergent differentiation showed frequent alterations in CDKN2A, CDKN2B, MTAP, CCND1, and the FGF19/FGF4/FGF3 amplicon, a profile relatively closer to that of PUC. These findings are consistent with previous studies reporting enrichment of TP53 and RB1 mutations in small cell variant UC [7], and lower frequencies of TERT promoter mutations in primary bladder adenocarcinoma [24]. Some of these subtype‐associated patterns, particularly in squamous differentiation, may reflect previously established molecular programs such as the basal/squamous subtype. Nevertheless, our findings extend these observations by linking routine histopathological classification with real‐world genomic profiling across multiple S/DD categories. However, additional subtype‐level analyses suggested that squamous and glandular differentiation may also possess distinct molecular features, indicating that divergent differentiation itself is not biologically uniform. Moreover, neuroendocrine tumors showed frequent TP53/RB1 alterations, whereas micropapillary tumors were enriched for ERBB2 alterations, further supporting subtype‐specific diversity. From a clinical perspective, this distinction may have important therapeutic implications. Co‐alterations of *RB1* and *TP53* have been associated with genomic biomarkers predictive of response to ICIs in muscle‐invasive bladder cancer [25]. Consistently, we observed significantly higher TMB levels in histological subtypes than in tumors with divergent differentiation, and S/DD was positively associated with PD‐L1 expression in our institutional cohort. Taken together, our findings emphasize the importance of histological context when interpreting genomic data in S/DD. Although some tumors with divergent differentiation may resemble PUC at a global genomic level, further studies are needed before reconsidering current pathological classification or treatment strategies.

Consistent with previous studies [1, 18], S/DD was significantly associated with adverse clinicopathological features and a poor prognosis in both BLCA and UTUC in the Hiroshima University cohort. We also demonstrated that S/DD was inversely associated with Nectin4 and TROP2 expression. Supporting this finding, reduced efficacy of enfortumab vedotin in variant histology has been reported [26]. We previously demonstrated that Nectin4 and TROP2 expression was associated with a favorable prognosis, luminal consensus, and the FGFR3 mutational subtype [15]. Antibody‐drug conjugates (ADC) combine the target specificity of monoclonal antibodies with the cytotoxicity of chemotherapy, and the clinical efficacy of enfortumab vedotin and sacituzumab govitecan have been demonstrated in both metastatic and locally advanced UC [16, 27]. However, the reduced expression of Nectin‐4 and TROP2 observed in S/DD suggests that the biological context of variant histology may influence the therapeutic effectiveness of ADC‐based approaches, as well as FGFR3‐targeted therapies. According to the current National Comprehensive Cancer Network guidelines [28], both enfortumab vedotin plus pembrolizumab and gemcitabine plus cisplatin with nivolumab are recommended as first‐line treatments for metastatic UC. Given that S/DD frequently exhibits low Nectin‐4 expression and basal‐like molecular features, platinum‐based chemotherapy combined with immune checkpoint blockade may be a rational strategy in this setting, as basal tumors tend to be more responsive to chemotherapy and immunotherapy [29]. However, our study was based on correlative molecular and immunohistochemical data and did not directly assess treatment response. Therefore, the optimal sequencing of immune checkpoint inhibitors and ADCs in S/DD should be evaluated in prospective clinical trials.

This study had several limitations. First, pathological classification in the C‐CAT dataset was based on institutional diagnoses without central review; therefore, interobserver variability and diagnostic heterogeneity cannot be excluded, particularly for histological subtypes/divergent differentiation. Second, the C‐CAT dataset lacks detailed clinical information, including TNM classification, disease stage, prior treatment, and drug response, limiting direct clinicogenomic correlations and stage‐specific interpretation of S/DD. Because the clinical significance and management of these subtypes differ substantially across NMIBC, MIBC, and locally advanced/metastatic disease settings, the absence of precise stage stratification may reduce the clinical interpretability of our findings. Third, oncoplot analyses were performed separately using the C‐CAT and MSK datasets. Although broadly similar genomic patterns were observed, several discrepancies were noted. For instance, *KMT2D* mutations were more frequent in PUC in the C‐CAT dataset, whereas they were more frequent in S/DD in the MSK dataset. KMT2D is known to be involved in the early events of carcinogenesis, and be frequently mutated in both UTUC and BLCA [30]. These differences may reflect variations in data structure, analytical approaches, and sequencing panel design and gene coverage (e.g., FoundationOne vs. MSK‐IMPACT), as well as potential population differences. Further studies using harmonized datasets are warranted. Finally, although additional stratified analyses of UTUC and BLCA were performed, C‐CAT includes patients with advanced solid malignancies who have completed or are expected to complete standard therapy, and therefore clinically complex or heavily pretreated cases may have been included. In addition, the number of UTUC cases with distinct histological subtypes was limited, and these findings should be interpreted cautiously.

In conclusion, we clarified the distribution of gene mutations in S/DD using a large‐scale nationwide genomic dataset of Japanese UC. S/DD was associated with distinct molecular features, including frequent TP53/RB1 alterations, low Nectin‐4 expression, and basal‐like characteristics, which may have implications for treatment selection. These findings provide a framework for a better biological understanding of S/DD and may inform the development of future therapeutic strategies.

## Author Contributions

**Go Kobayashi:** conceptualization, methodology, investigation, visualization, writing – original draft, formal analysis, data curation, software, project administration. **Shunsuke Miyamoto:** writing – review and editing. **Keisuke Goto:** writing – review and editing. **Yohei Sekino:** writing – review and editing, methodology, data curation, project administration, supervision. **Hiroyuki Kitano:** writing – review and editing. **Akihiro Goriki:** writing – review and editing. **Kazuhiro Sentani:** writing – review and editing. **Testutaro Hayashi:** writing – review and editing. **Kohei Kobatake:** writing – review and editing. **Nobuyuki Hinata:** writing – review and editing. **Keisuke Hieda:** writing – review and editing.

## Funding

This research did not receive any specific grant from funding agencies in the public, commercial, or not‐for‐profit sectors.

## Ethics Statement

This retrospective study was approved by the Ethics Committee at Hiroshima University (authorization number: E20001‐9923). This investigation was conducted in accordance with the Declaration of Helsinki of 1975.

## Consent

Informed consent was obtained from each patient.

## Conflicts of Interest

Go Kobayashi, Yohei Sekino, Shunsuke Miyamoto, Kohei Kobatake, Hiroyuki Kitano, Keisuke Goto, Akihiro Goriki, Keisuke Hieda, Testutaro Hayashi, Kazuhiro Sentani declare no conflicts of interest. Nobuyuki Hinata is an Editorial Board member of the International Journal of Urology and a coauthor of this article. To minimize bias, he was excluded from all editorial decision‐making processes related to the acceptance of this article for publication.

## Supporting information


**Data S1:** Supplementary Methods S1. Details of patient information and the immunohistochemical analysis of various molecules.

**Data S2:** Supplementary Methods S2. The Python codes used in this study are presented.

**Figure S1:** Genomic variables of urothelial carcinoma subtypes/divergent differentiation (S/DD) and pure urothelial carcinoma (PUC). Comparison of the numbers of (A) somatic mutations, (B) copy number alterations (CNAs), and (C) gene rearrangements between S/DD and PUC in the Center for Cancer Genomics and Advanced Therapeutics dataset. *`* values were calculated using Mann–Whitney U test, with statistically significant differences highlighted in red. Asterisks indicate statistical significance (*p < 0.05; **p < 0.01).

**Figure S2:** Genomic profiles of urothelial carcinoma with subtypes/divergent differentiation (S/DD) and pure urothelial carcinoma (PUC), stratified by upper tract urothelial carcinoma (UTUC) and bladder urothelial carcinoma (BLCA) in the C‐CAT dataset. Oncoplots showing somatic mutations in frequently altered genes are presented for (A) S/DD (*n* = 166) and (B) PUC (*n* = 612) in BLCA, and (C) S/DD (*n* = 43) and (D) PUC (*n* = 448) in UTUC. Oncoplots of copy number alterations (CNAs) are shown for (E) S/DD (*n* = 121) and (F) PUC (*n* = 493) in BLCA, and (G) S/DD (*n* = 34) and (H) PUC (*n* = 328) in UTUC.

**Figure S3:** Genomic profiles of urothelial carcinoma subgroups defined by histological subtypes and divergent differentiation, stratified by upper tract urothelial carcinoma (UTUC) and bladder urothelial carcinoma (BLCA) in the C‐CAT dataset. Oncoplots showing somatic mutations in frequently altered genes are presented for (A) histological subtypes (*n* = 90) and (B) divergent differentiation (*n* = 76) in BLCA, and (C) histological subtypes (*n* = 13) and (D) divergent differentiation (*n* = 30) in UTUC. Oncoplots of copy number alterations (CNAs) are shown for (E) histological subtypes (*n* = 64) and (F) divergent differentiation (*n* = 57) in BLCA, and (G) histological subtypes (*n* = 11) and (H) divergent differentiation (*n* = 23) in UTUC.

**Figure S4:** Genomic profiles of urothelial carcinoma with subtypes/divergent differentiation (S/DD), stratified by each histological subtype, in the C‐CAT dataset. Oncoplots showing somatic mutations in frequently altered genes are presented for (A) squamous differentiation (*n* = 60), (B) glandular differentiation (*n* = 47), (C) neuroendocrine carcinoma (*n* = 71), (D) plasmacytoid/signet ring cell carcinoma (*n* = 18), (E) micropapillary carcinoma (*n* = 9), and (F) other histological subtypes (*n* = 14).

**Figure S5:** Genomic profiles of urothelial carcinoma with subtypes/divergent differentiation (S/DD), stratified by each histological subtype, in the C‐CAT dataset. Oncoplots showing copy number alterations (CNAs) in frequently altered genes are presented for (A) squamous differentiation (*n* = 47), (B) glandular differentiation (*n* = 34), (C) neuroendocrine carcinoma (*n* = 53), (D) plasmacytoid/signet ring cell carcinoma (*n* = 12), (E) micropapillary carcinoma (*n* = 6), and (F) other histological subtypes (*n* = 10).

**Figure S6:** Prognostic impact of urothelial carcinoma subtypes/divergent differentiation (S/DD) on clinical outcomes. Kaplan–Meier survival curves comparing S/DD and pure urothelial carcinoma (PUC). (A) Overall survival (OS) in the MSK2022 dataset. (B) Cancer‐specific survival (CSS) and (C) progression‐free survival (PFS) in upper tract urothelial carcinoma (UTUC) in the Hiroshima University cohort. (D) CSS in bladder urothelial carcinoma (BLCA) in the Hiroshima University cohort. *p* values were calculated using Log rank test, with statistically significant differences highlighted in red. Asterisks indicate statistical significance (*p < 0.05; **p < 0.01).

**Figure S7:** Representative immunohistochemical staining patterns of markers associated with histological subtypes/divergent differentiation (S/DD) in upper tract urothelial carcinoma. Representative images of markers positively associated with S/DD are shown in the left panel, including PD‐L1, EGFR, and p53. Representative images of markers inversely associated with S/DD are shown in the right panel, including FGFR3, GATA3, Nectin‐4, and TROP2. Scale bars = 50 μm.

**Table S1:** Summary of frequently mutated genes in subtypes/divergent differentiation and pure urothelial carcinoma in C‐CAT database.

**Table S2:** Summary of frequently mutated genes in subtypes/divergent differentiation and pure urothelial carcinoma in MSK2022 database.

**Table S3:** Summary of frequently mutated genes in histological subtypes and divergent differentiation in C‐CAT database.

**Table S4:** Summary of frequently mutated genes in histological subtypes and divergent differentiation in MSK2022 database.

**Table S5:** Comparison of each urothelial carcinoma subtypes/divergent differentiation (S/DD) and pure urothelial carcinoma.

## Data Availability

The data that support the findings of this study are available from the corresponding author upon reasonable request.
