# Supplementary material for: Comprehensive Genomic Characterization Between Urothelial Carcinoma Subtypes/Divergent Differentiation (S/DD) and Pure Urothelial Carcinoma Using a Large‐Scale Japanese Genomic Panel Dataset
Source: Int J Urol. 2026 Jun 8;33(6):e70538. doi: 10.1111/iju.70538 (PMC13244187; doi:10.1111/iju.70538)
Supplement: Supplementary file 1 — Data S1: Supplementary Methods S1. Details of patient information and the immunohistochemical analysis of various molecules. [file IJU-33-0-s001.docx]

**Detailed information of the C-CAT and MSK datasets**

Data from nearly all targeted sequencing tests performed using the FoundationOne® CDx and NCC Oncopanel under Japan’s national health insurance system have been collected by the C-CAT, with written informed consent obtained from each patient. The use of C-CAT data was approved by the C-CAT Information Utilization Review Board (CDU2024-028N). Between June 2019 and April 2025, 102,424 comprehensive cancer genomic profiling (CGP) results from advanced solid tumors across 30 organs were registered in the database. In Japan, CGP testing is reimbursed only for patients with advanced solid malignancies who have completed or are expected to complete standard-of-care therapy.

Among these, 1,292 UC cases (BLCA and UTUC) were histologically classified as PUC (n = 1,073) or S/DD (n = 219) based on pathological diagnosis (Table 1). According to the 2022 ISUP Consensus Conference recommendations, S/DD cases were further categorized into divergent differentiation (n = 108) and distinct histological subtypes (n = 111) (Table 2). Cases of urachal carcinoma were excluded. Genomic and clinical data were extracted from the C-CAT database for retrospective analysis. Additional subgroup analyses were performed in the C-CAT cohort after separating UTUC and BLCA cases. Among the 1,292 total cases, 494 were classified as UTUC and 783 as BLCA; cases with unclear primary origin were excluded (n = 15).

We additionally analyzed publicly available genomic data from the MSK-IMPACT assay (MSK2022 dataset). Among 1,656 UC samples, 1,621 were classified as PUC and 35 as S/DD based on the “Cancer Type Detailed” category (Table 1). Urachal carcinoma and cancers of unknown primary were excluded. Similar to the C-CAT analysis, genomic alterations were compared between S/DD and PUC, as well as between divergent differentiation (n = 14) and distinct histological subtypes (n = 21) (Table 2). Because different sequencing panels were used, the MSK and C-CAT cohorts were analyzed separately, with the MSK dataset used for descriptive comparison.

**Patients with UTUC and BLCA in Hiroshima university cohort**

The medical records of patients who underwent radical nephroureterectomy for unilateral UTUC at Hiroshima University Hospital between April 1999 and May 2019 were retrospectively reviewed. Patients who received neoadjuvant chemotherapy were excluded from this study. Pathology specimens were examined and re-reviewed for staging according to the 8th edition of the American Joint Committee on Cancer/Union for International Cancer Control (AJCC/UICC) TNM classification (2017). We used the 2004 WHO/ISUP 2-tier grading system to evaluate the tumor grade. The study endpoints were CSS and PFS, with progression defined as lymph node relapse or distant metastasis (excluding bladder cancer recurrence or contralateral UTUC). Follow-up included urinalysis and chest-abdomen-pelvis CT every 3–6 months for at least five years, per physician preference.

Patients who were diagnosed with BC and treated with radical cystectomy (RC) at Hiroshima University Hospital or affiliated hospitals in 1995–2015 were included if they did not undergo neoadjuvant chemotherapy. We collected tumor samples from 93 patients who had undergone RC. Tumor staging and TNM pathological classification were conducted according to the 1973 WHO International Society of Urological Pathology Consensus classification and the UICC TNM Classification of Malignant Tumors (7th edition).

**Immunohistochemical analysis for various cancer related molecules**

Immunohistochemical analyses were performed for the following cancer-related markers: Ki-67 (1:400, mouse monoclonal, clone M7240; Dako, Agilent Technologies, Santa Clara, CA, USA), PD-L1 (1:300, rabbit monoclonal, clone 28–8; Abcam, Cambridge, UK), CD8 (1:50, mouse monoclonal, clone 4B11; Leica Biosystems, Wetzlar, Germany), HER2 (1:200, rabbit polyclonal, clone A0485; Dako, Agilent Technologies, Santa Clara, CA, USA), EGFR (1:25, mouse monoclonal, clone EGFR113; Leica Biosystems, Wetzlar, Germany), FGFR3 (1:100, rabbit monoclonal, clone C51F2; Cell Signaling Technology, Danvers, MA, USA), p53 (1:200, mouse monoclonal, clone DO-7; Leica Biosystems, Wetzlar, Germany), GATA3 (ready-to-use, mouse monoclonal, clone L50-823; Nichirei Biosciences Inc., Tokyo, Japan), UPK3 (ready-to-use, mouse monoclonal, clone AU1; Nichirei Biosciences Inc., Tokyo, Japan), CK5/6 (1:200, mouse monoclonal, clone D5/16B4; Dako, Agilent Technologies, Santa Clara, CA, USA), Nectin4 (1:1000, rabbit monoclonal, clone EPR15613-68; Abcam, Cambridge, UK) and TROP2 (1:100, goat polyclonal, AF650; R&D Systems, Inc., Minneapolis, MN, USA).

All markers were evaluated independently by three investigators (G.K., T.H., K.S.) and scored as positive or negative according to predefined criteria. Positivity was defined as follows: >20% of tumor cells showing nuclear staining for Ki-67 and GATA3; continuous staining across all epithelial layers for CK5/6; positive staining in >10% of tumor cells for p53; and positive staining in >10% of tumor-infiltrating lymphocytes (TILs) for CD8. UPK3 expression was considered positive when any tumor cell showed immunoreactivity. HER2, EGFR, and FGFR3 expression was scored based on staining intensity using a four-tier system (0 = no staining, 1+ = weak staining, 2+ = moderate staining, 3+ = strong staining). Tumors with an intensity score of 2+ or 3+ were considered positive. PD-L1 expression was evaluated on the membranes of tumor cells (TCs) and tumor-infiltrating lymphocytes (TILs). Positivity was defined as membranous staining in ≥1% of cells, using median cutoff values for TCs and TILs, respectively. Nectin4 expression was assessed based on membranous and/or cytoplasmic staining. Tumors were classified as Nectin4-positive when more than 25% of tumor cells showed positive staining. TROP2 expression levels were quantified using the H-score, and specimens were classified into TROP2 positive (H-score ≥ 100) and negative (H-score < 100).
