# Supplementary material for: Comprehensive Genomic Characterization Between Urothelial Carcinoma Subtypes/Divergent Differentiation (S/DD) and Pure Urothelial Carcinoma Using a Large‐Scale Japanese Genomic Panel Dataset
Source: Int J Urol. 2026 Jun 8;33(6):e70538. doi: 10.1111/iju.70538 (PMC13244187; doi:10.1111/iju.70538)
Supplement: Supplementary file 2 — Data S2: Supplementary Methods S2. The Python codes used in this study are presented. [file IJU-33-0-s009.docx]

**# Python code for generating an oncoplot**

import pandas as pd

import matplotlib.pyplot as plt

import seaborn as sns

# Load the data

data = pd.read_csv("File name.csv", low_memory=False)

# Remove rows with missing key information

data = data.dropna(subset=["Sample ID", "Variant", "Hugo_Symbol", "Variant_Classification"])

# Split data into variant and pure (common) groups

Variant = data[data["Variant"] == "variant"]

Pure = data[data["Variant"] == "common"]

# Display the number of samples in each group

print("Variant group:", Variant["Sample ID"].nunique(), "samples")

print("Common group:", Pure["Sample ID"].nunique(), "samples")

# Function to plot Oncoplot with mutation counts

def plot_oncoplot_with_bars(data, title, filename):

# Group mutation information by gene, sample, and mutation type

mutation_matrix = data.groupby(["Hugo_Symbol", "Sample ID", "Variant_Classification"]).size().reset_index(name="Count")

# Extract top 20 most frequently mutated genes based on the number of samples

top_genes = (

mutation_matrix.groupby("Hugo_Symbol")["Sample ID"]

.nunique()

.sort_values(ascending=False)

.head(20)

.index

)

mutation_matrix = mutation_matrix[mutation_matrix["Hugo_Symbol"].isin(top_genes)]

# Create a pivot table for visualization

pivot_table = mutation_matrix.pivot_table(

index="Hugo_Symbol", columns="Sample ID", values="Variant_Classification", aggfunc="first"

)

# Calculate mutation counts per sample and per gene

sample_mutation_counts = pivot_table.notna().sum(axis=0)

gene_mutation_counts = pivot_table.notna().sum(axis=1)

# Sort genes by mutation frequency (descending)

sorted_genes = gene_mutation_counts.sort_values(ascending=False).index

pivot_table = pivot_table.loc[sorted_genes]

gene_mutation_counts = gene_mutation_counts.loc[sorted_genes]

# Assign colors to mutation types

mutation_types = mutation_matrix["Variant_Classification"].dropna().unique()

color_palette = sns.color_palette("tab10", len(mutation_types))

color_map = {

"Missense_Mutation": "#1f77b4", # blue

"Nonsense_Mutation": "#ff7f0e", # orange

"Frameshift_Mutation": "#d62728", # red

"Splice site_Mutation": "#2ca02c" # green

}

# Generate color map for tiles

tile_colors = pivot_table.applymap(lambda x: color_map.get(x, "white"))

# Create figure

fig = plt.figure(figsize=(17, 13))

num_genes = len(pivot_table.index)

num_samples = len(pivot_table.columns)

# Define grid layout

gs = fig.add_gridspec(

3, 2,

width_ratios=[1, 0.3],

height_ratios=[1, len(pivot_table.index), 0.5],

hspace=0.05, wspace=0.11

)

# Total number of samples

total_samples = len(data["Sample ID"].unique())

# Calculate mutation counts and frequency (%)

gene_mutation_counts = pivot_table.notna().sum(axis=1)

gene_mutation_percent = (gene_mutation_counts / total_samples * 100).round(1)

# Sort order

sorted_genes = gene_mutation_counts.sort_values(ascending=False).index

pivot_table = pivot_table.loc[sorted_genes]

gene_mutation_counts = gene_mutation_counts.loc[sorted_genes]

gene_mutation_percent = gene_mutation_percent.loc[sorted_genes]

# Top bar plot (Mutation Count per sample)

ax_bar_top = fig.add_subplot(gs[0, 0])

ax_bar_top.bar(range(num_samples), sample_mutation_counts.values, color="gray", align="center", width=1.0)

ax_bar_top.set_xlim(-0.5, num_samples - 0.5)

ax_bar_top.set_ylabel("Mutation Count", fontsize=8)

ax_bar_top.set_ylim(0, 20)

ax_bar_top.set_xticks([])

ax_bar_top.spines["right"].set_visible(False)

ax_bar_top.spines["top"].set_visible(False)

# Right bar plot (Gene frequency)

ax_bar_right = fig.add_subplot(gs[1, 1])

ax_bar_right.barh(range(num_genes), gene_mutation_counts.values, color="gray", align="center", height=1.0)

ax_bar_right.set_ylim(-0.5, num_genes - 0.5)

ax_bar_right.set_xlabel("Gene Frequency", fontsize=10)

ax_bar_right.set_yticks(range(num_genes))

ax_bar_right.set_yticklabels([f"{perc}%" for perc in gene_mutation_percent], fontsize=11)

ax_bar_right.invert_yaxis()

ax_bar_right.spines["right"].set_visible(False)

ax_bar_right.spines["top"].set_visible(False)

# Main oncoplot (mutation tiles)

ax_main = fig.add_subplot(gs[1, 0])

for i, row in enumerate(tile_colors.iterrows()):

for j, color in enumerate(row[1]):

ax_main.add_patch(plt.Rectangle((j, i), 1, 1, color=color))

ax_main.set_xlim(0, len(pivot_table.columns))

ax_main.set_ylim(0, len(pivot_table.index))

ax_main.set_xticks([x + 0.5 for x in range(len(pivot_table.columns))])

ax_main.set_xticklabels([])

ax_main.set_yticks([y + 0.5 for y in range(len(pivot_table.index))])

ax_main.set_yticklabels(pivot_table.index, fontsize=11)

ax_main.invert_yaxis()

# Legend

ax_legend = fig.add_subplot(gs[2, 0])

legend_elements = [

plt.Line2D([0], [0], color=color, lw=4, label=mutation_type)

for mutation_type, color in color_map.items()

]

ax_legend.legend(

handles=legend_elements, loc="center", ncol=5, fontsize=10, frameon=False, bbox_to_anchor=(0.5, -0.001)

)

ax_legend.axis("off")

# Final adjustments

fig.suptitle(title, fontsize=14, y=0.95)

plt.tight_layout()

# Save figure (PNG, 300 dpi)

fig.savefig(filename, dpi=300, bbox_inches="tight")

plt.show()

# Generate plots for both groups

plot_oncoplot_with_bars(Variant, "Oncoplot with Bars: Variant Group", "variant_group_Oncoplot.png")

plot_oncoplot_with_bars(Pure, "Oncoplot with Bars: Common Group", "common_group_Oncoplot.png")

# Extract all mutated genes

data = pd.read_csv("File name.csv", low_memory=False)

# Remove rows with missing key information

data = data.dropna(subset=["Sample ID", "Variant", "Hugo_Symbol", "Variant_Classification"])

# Select target group (Variant group)

Variant = data[data["Variant"] == "variant"]

# Total number of samples in the variant group

total_samples = Variant["Sample ID"].nunique()

# Count the number of mutated samples per gene

mutation_counts = (

Variant.groupby("Hugo_Symbol")["Sample ID"]

.nunique()

.reset_index()

.rename(columns={"Sample ID": "Mutated_Samples"})

)

# Calculate mutation frequency (%)

mutation_counts["Mutation_Percentage"] = (mutation_counts["Mutated_Samples"] / total_samples * 100).round(2)

# Sort genes by mutation frequency (descending)

mutation_counts = mutation_counts.sort_values(by="Mutation_Percentage", ascending=False)

# Export results to CSV

mutation_counts.to_csv("File name.csv", index=False)

# Display top 5 genes

print(mutation_counts.head())
