# Supplementary figures and images for: Comprehensive Genomic Characterization Between Urothelial Carcinoma Subtypes/Divergent Differentiation (S/DD) and Pure Urothelial Carcinoma Using a Large‐Scale Japanese Genomic Panel Dataset
Source: Int J Urol. 2026 Jun 8;33(6):e70538. doi: 10.1111/iju.70538 (PMC13244187; doi:10.1111/iju.70538)

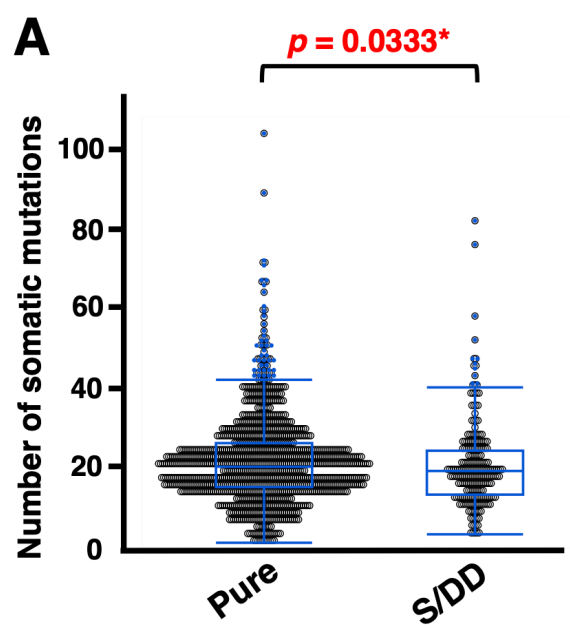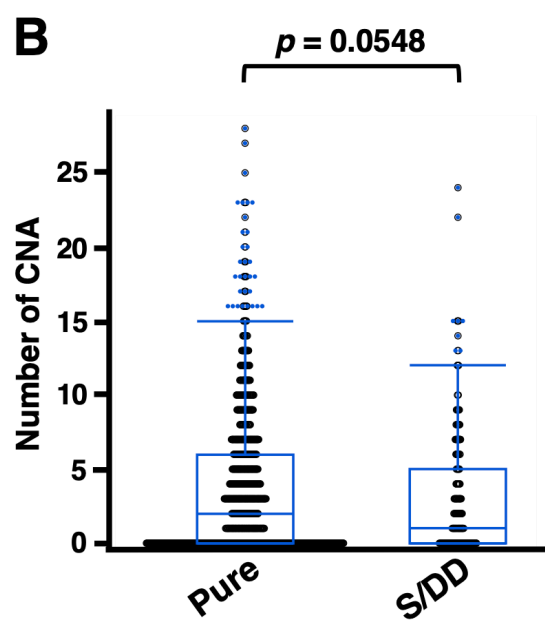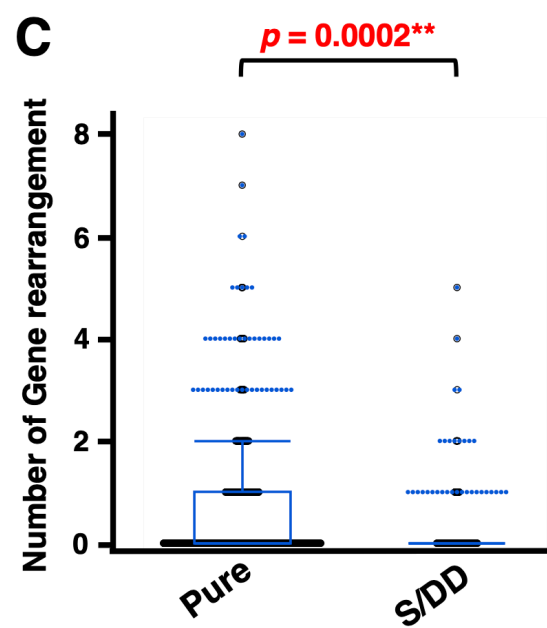

Supplement: Supplementary file 3 — Figure S1: Genomic variables of urothelial carcinoma subtypes/divergent differentiation (S/DD) and pure urothelial carcinoma (PUC). Comparison of the numbers of (A) somatic mutations, (B) copy number alterations (CNAs), and (C) gene rearrangements between S/DD and PUC in the Center for Cancer Genomics and Advanced Therapeutics dataset. ` values were calculated using Mann–Whitney U test, with statistically significant differences highlighted in red. Asterisks indicate statistical significance (*p < 0.05; **p < 0.01). [file IJU-33-0-s014.pdf]

# C-CAT database

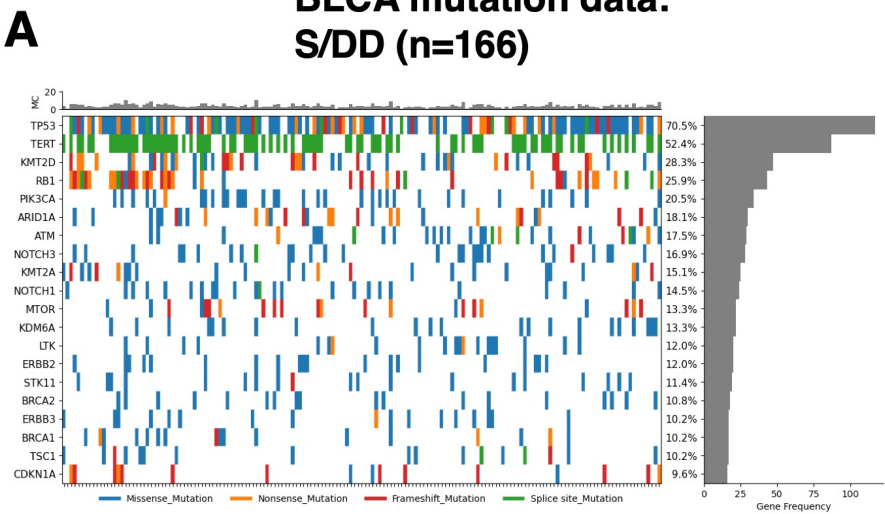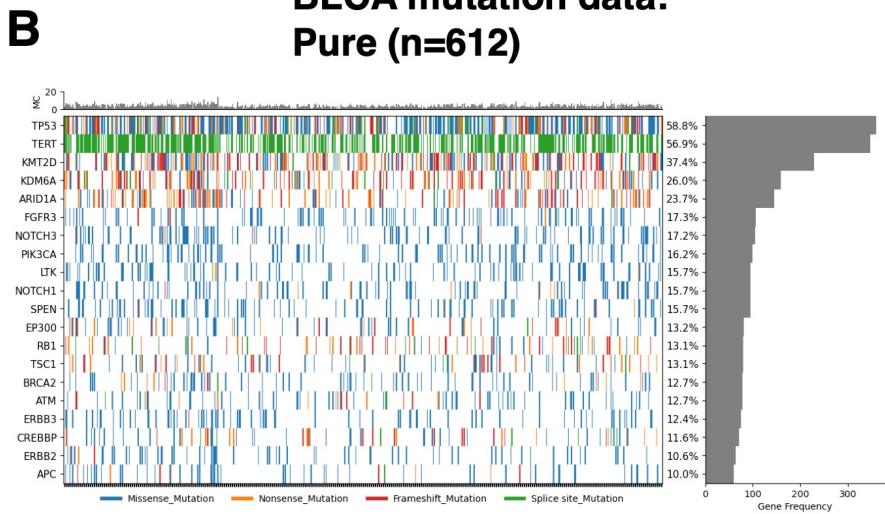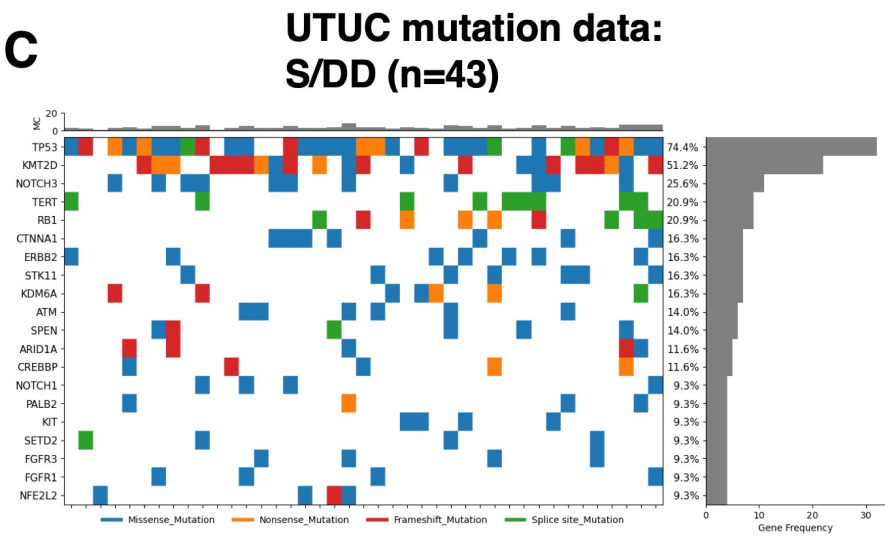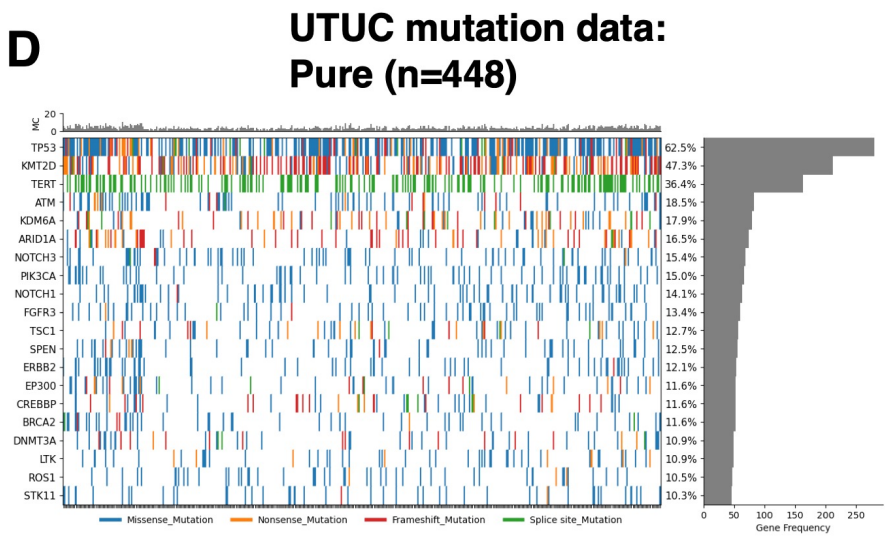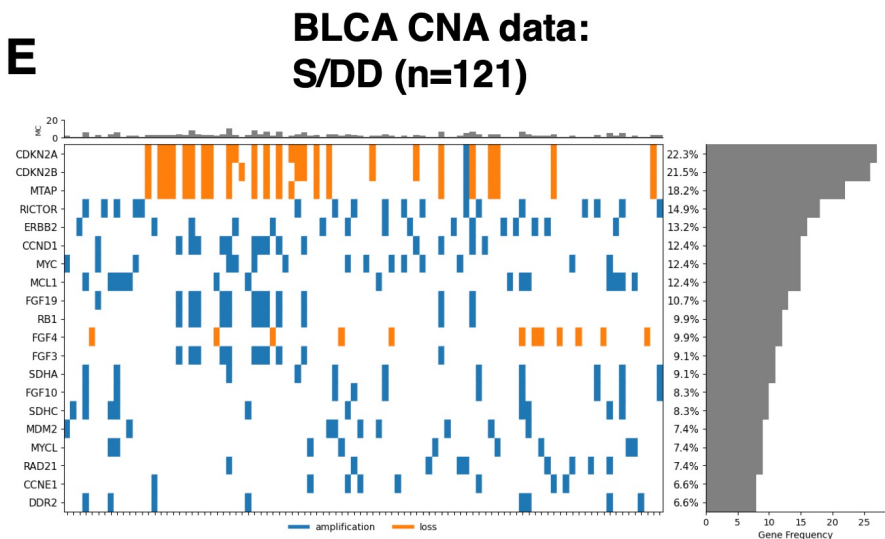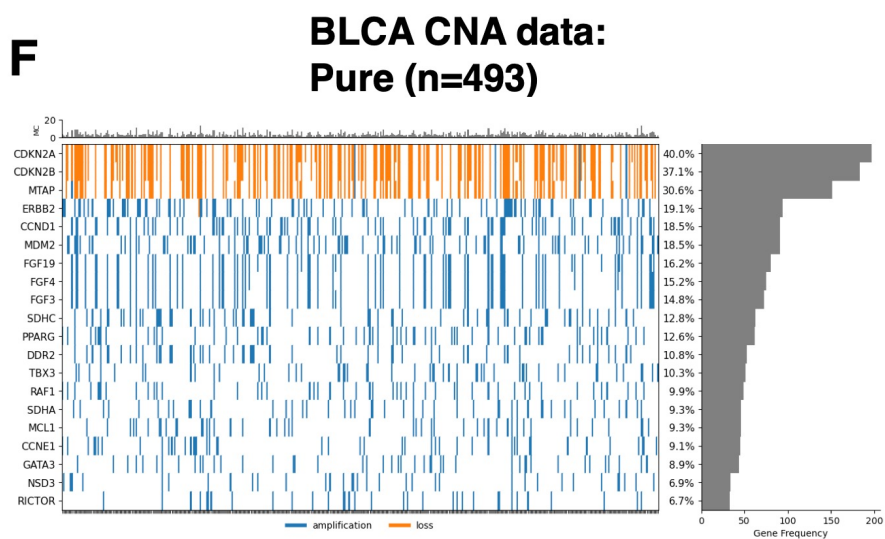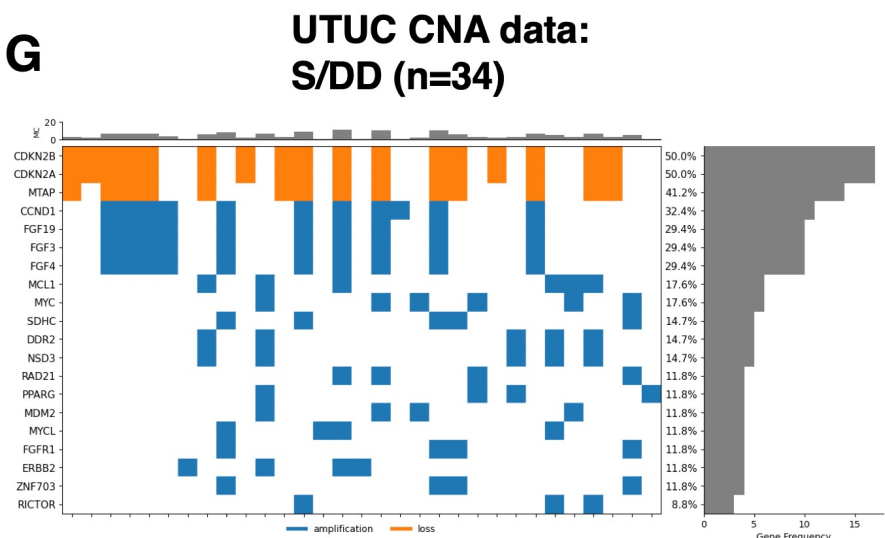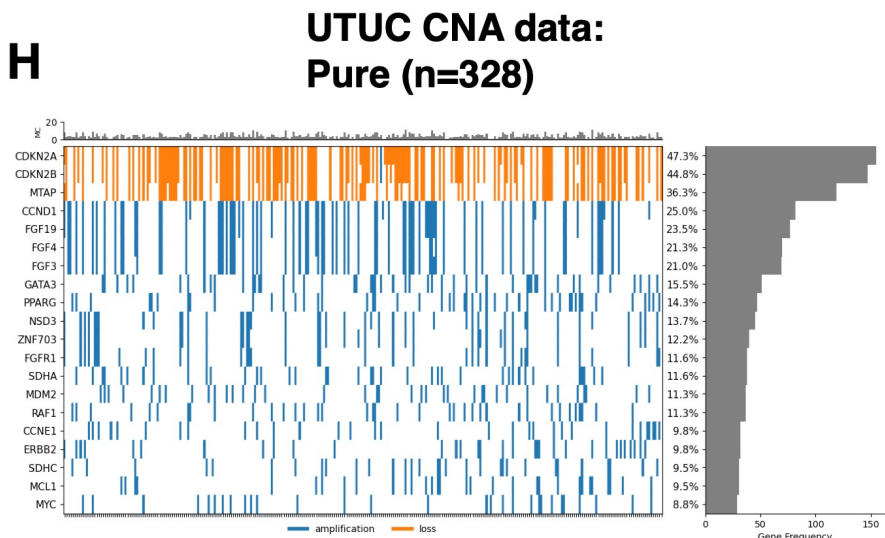

Supplement: Supplementary file 4 — Figure S2: Genomic profiles of urothelial carcinoma with subtypes/divergent differentiation (S/DD) and pure urothelial carcinoma (PUC), stratified by upper tract urothelial carcinoma (UTUC) and bladder urothelial carcinoma (BLCA) in the C‐CAT dataset. Oncoplots showing somatic mutations in frequently altered genes are presented for (A) S/DD (n = 166) and (B) PUC (n = 612) in BLCA, and (C) S/DD (n = 43) and (D) PUC (n = 448) in UTUC. Oncoplots of copy number alterations (CNAs) are shown for (E) S/DD (n = 121) and (F) PUC (n = 493) in BLCA, and (G) S/DD (n = 34) and (H) PUC (n = 328) in UTUC. [file IJU-33-0-s013.pdf]

# C-CAT database

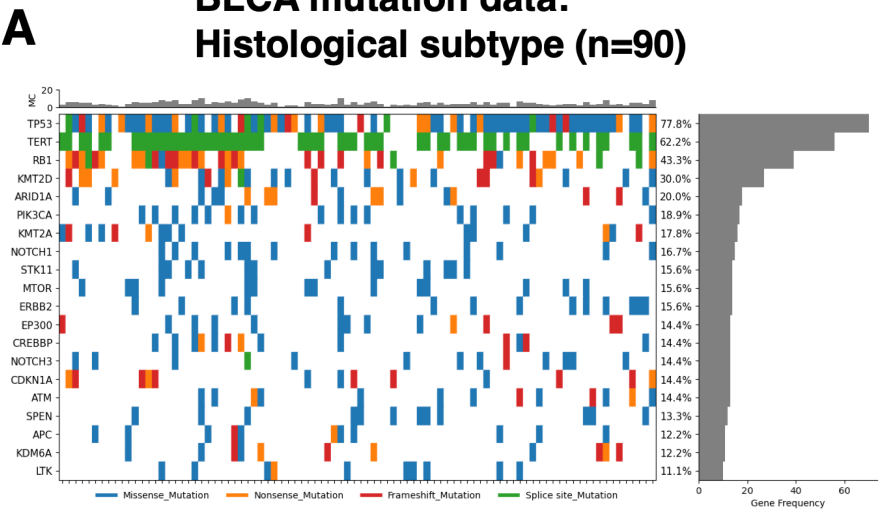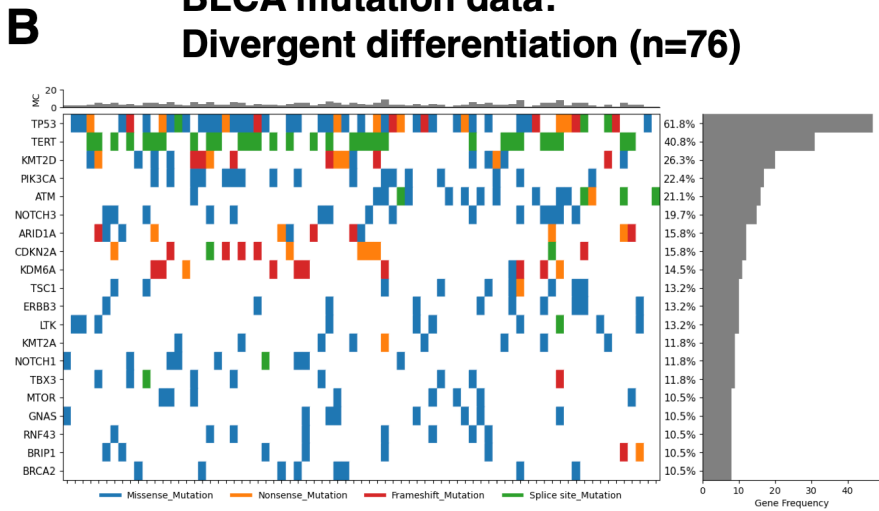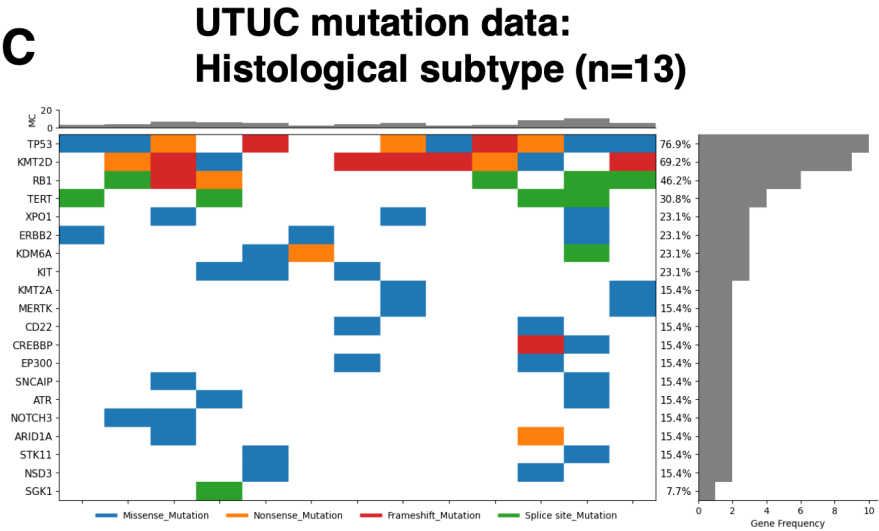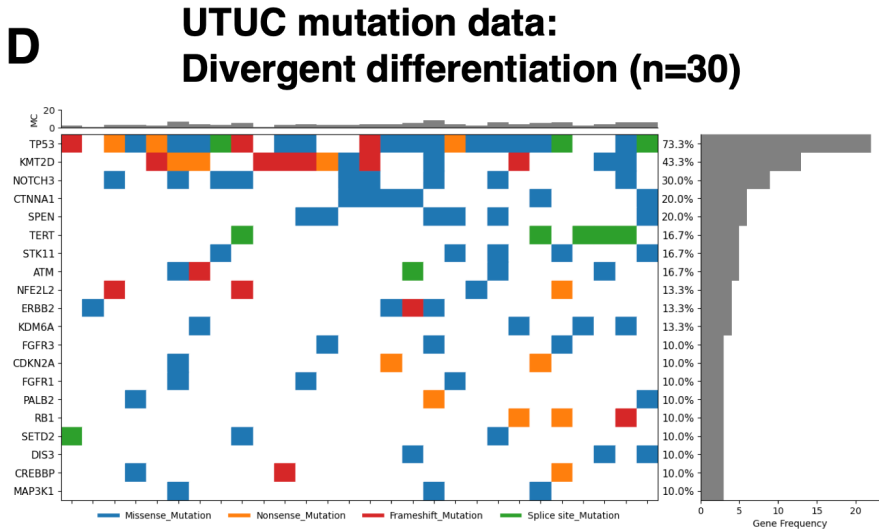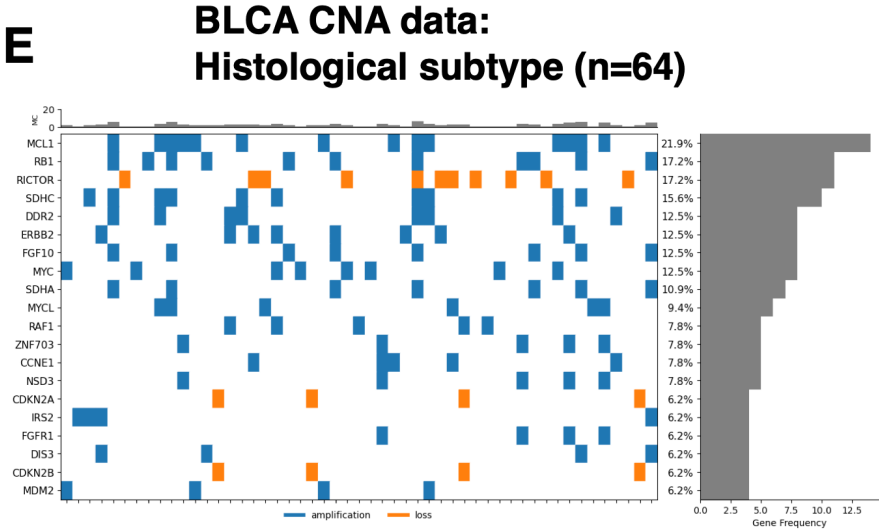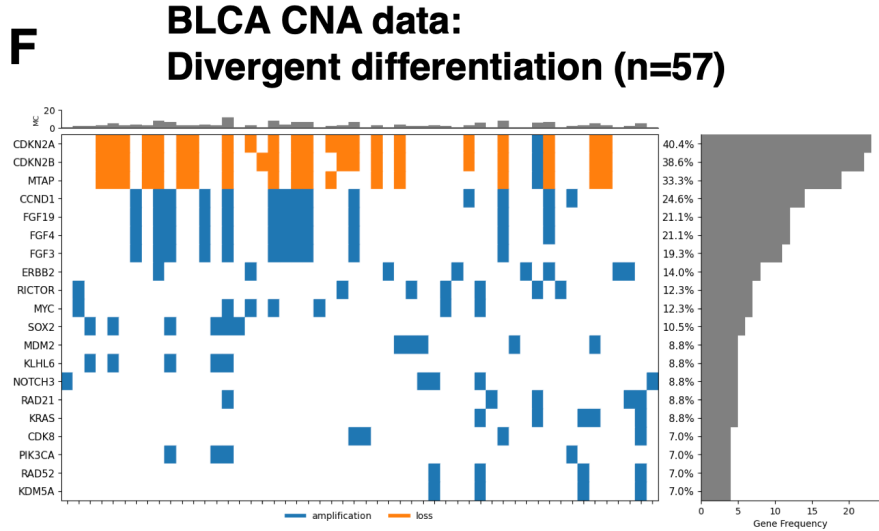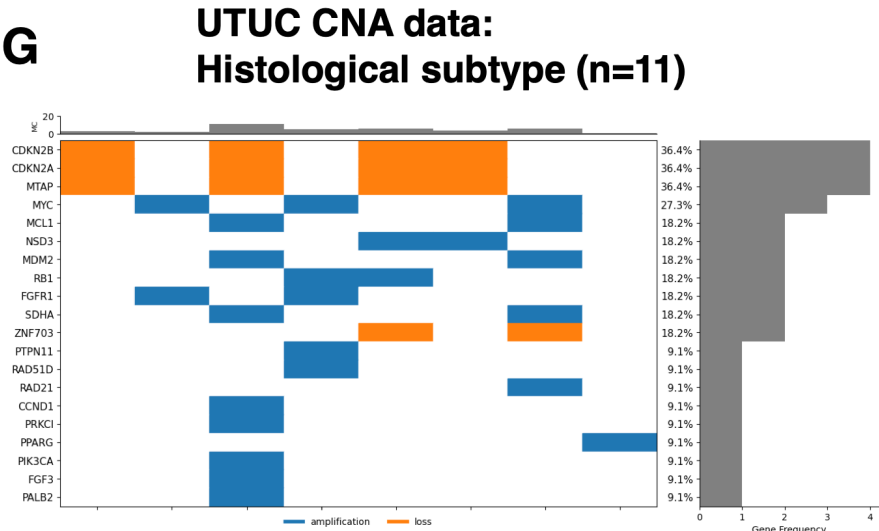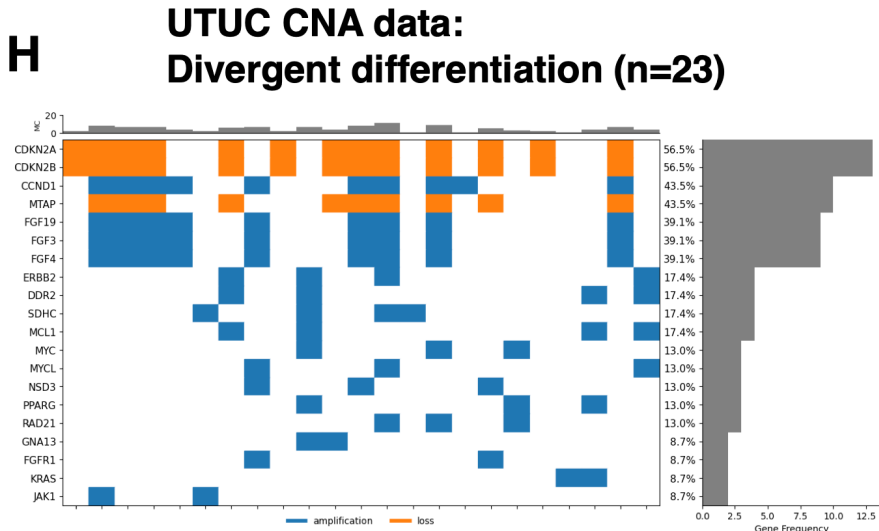

Supplement: Supplementary file 5 — Figure S3: Genomic profiles of urothelial carcinoma subgroups defined by histological subtypes and divergent differentiation, stratified by upper tract urothelial carcinoma (UTUC) and bladder urothelial carcinoma (BLCA) in the C‐CAT dataset. Oncoplots showing somatic mutations in frequently altered genes are presented for (A) histological subtypes (n = 90) and (B) divergent differentiation (n = 76) in BLCA, and (C) histological subtypes (n = 13) and (D) divergent differentiation (n = 30) in UTUC. Oncoplots of copy number alterations (CNAs) are shown for (E) histological subtypes (n = 64) and (F) divergent differentiation (n = 57) in BLCA, and (G) histological subtypes (n = 11) and (H) divergent differentiation (n = 23) in UTUC. [file IJU-33-0-s008.pdf]

MSK2022 database (n=1657)

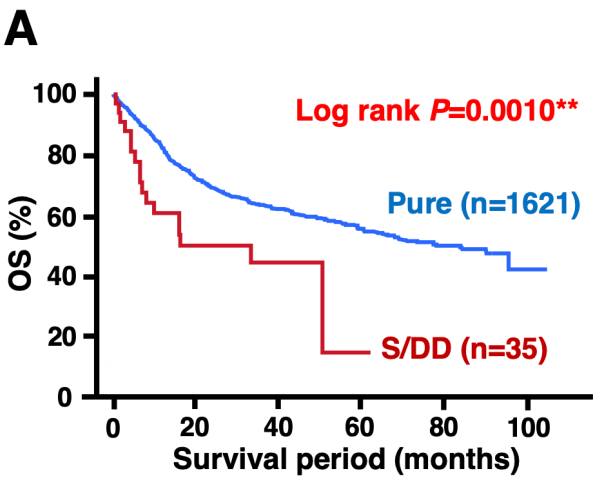

Hiroshima cohort

UTUC (n=153)

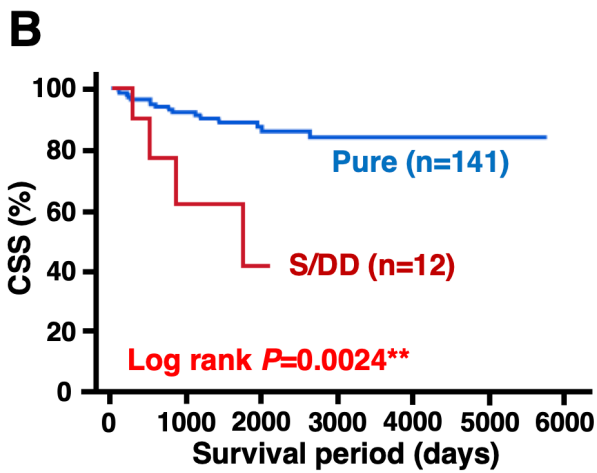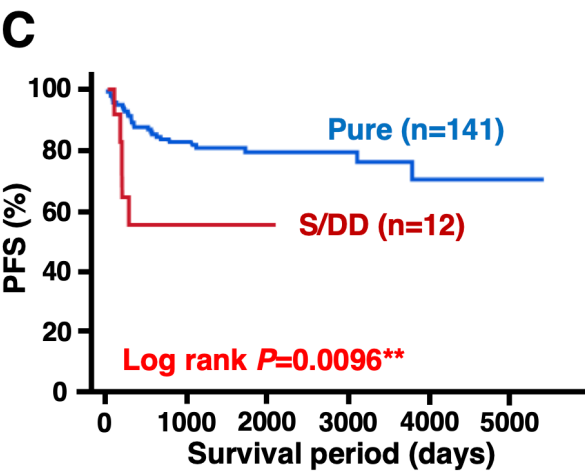

BLCA (n=93)

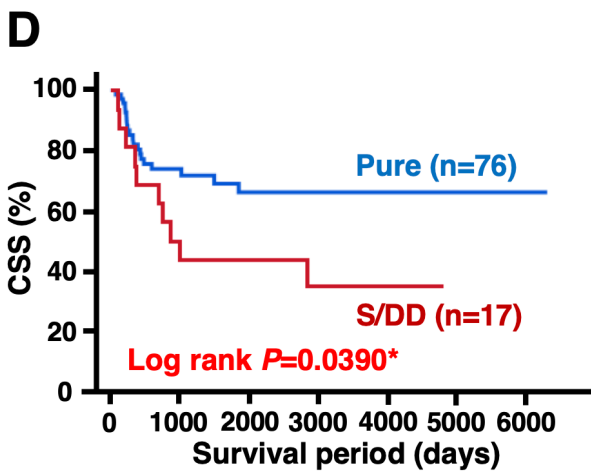

Supplement: Supplementary file 8 — Figure S6: Prognostic impact of urothelial carcinoma subtypes/divergent differentiation (S/DD) on clinical outcomes. Kaplan–Meier survival curves comparing S/DD and pure urothelial carcinoma (PUC). (A) Overall survival (OS) in the MSK2022 dataset. (B) Cancer‐specific survival (CSS) and (C) progression‐free survival (PFS) in upper tract urothelial carcinoma (UTUC) in the Hiroshima University cohort. (D) CSS in bladder urothelial carcinoma (BLCA) in the Hiroshima University cohort. p values were calculated using Log rank test, with statistically significant differences highlighted in red. Asterisks indicate statistical significance (*p < 0.05; **p < 0.01). [file IJU-33-0-s011.pdf]

**Markers positively associated  
with S/DD**

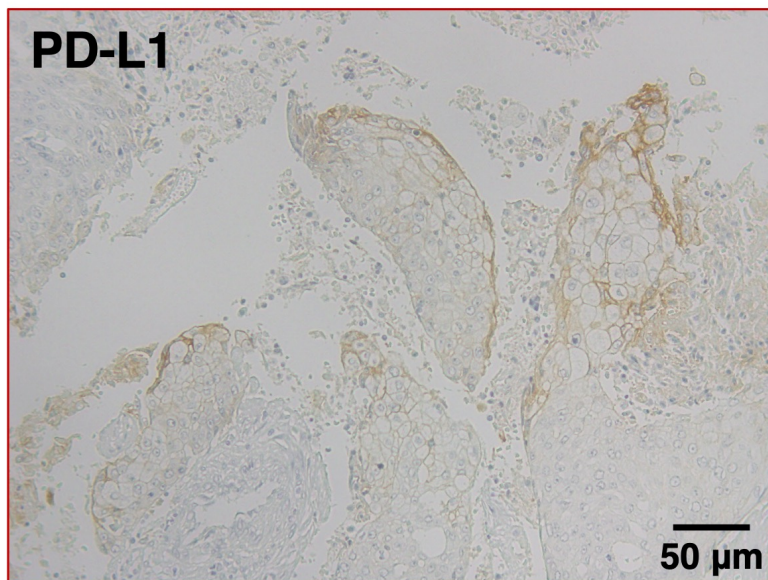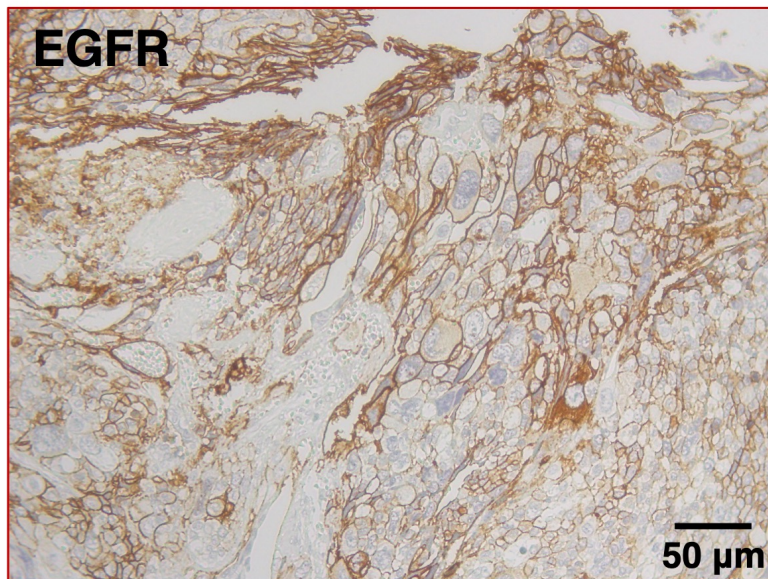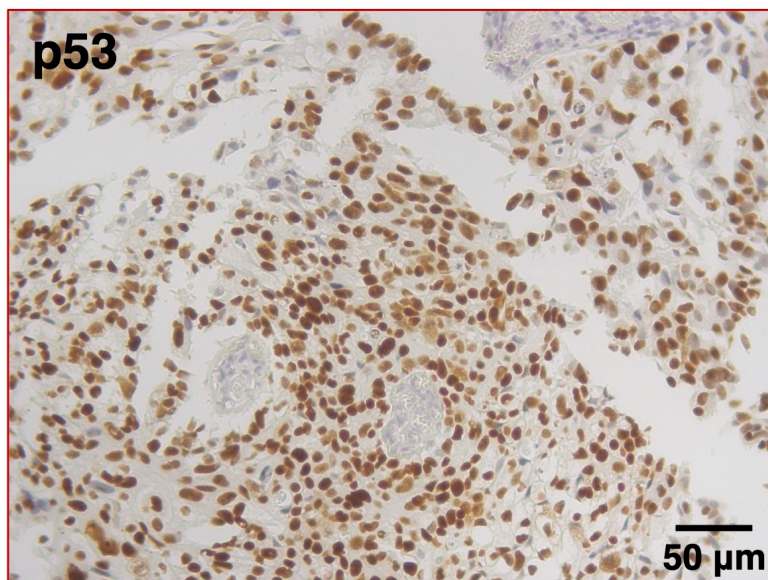

**Markers inversely associated  
with S/DD**

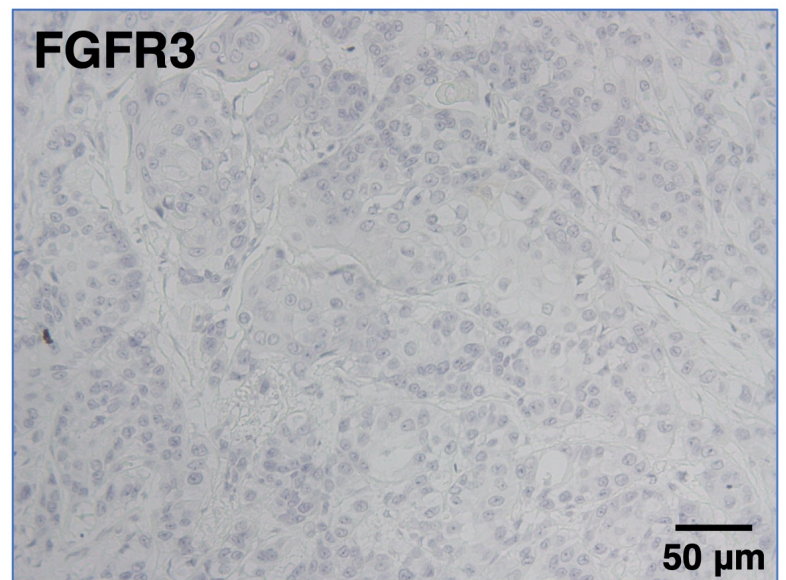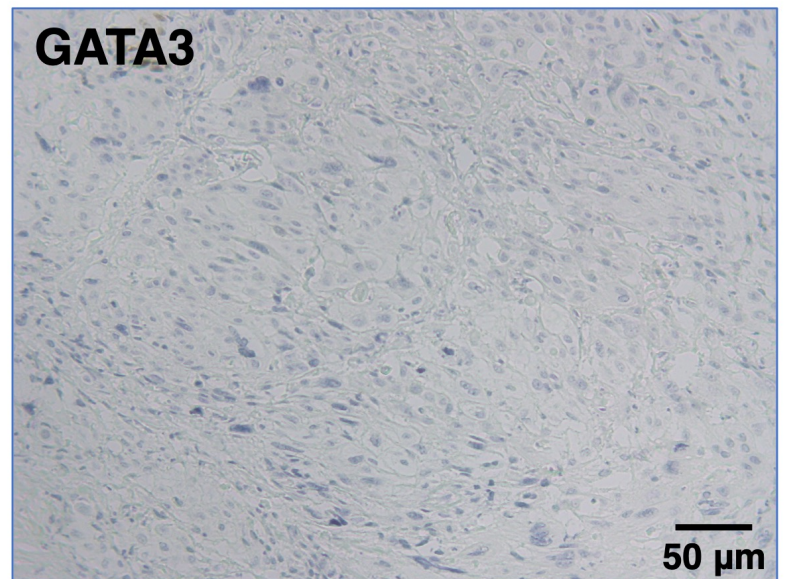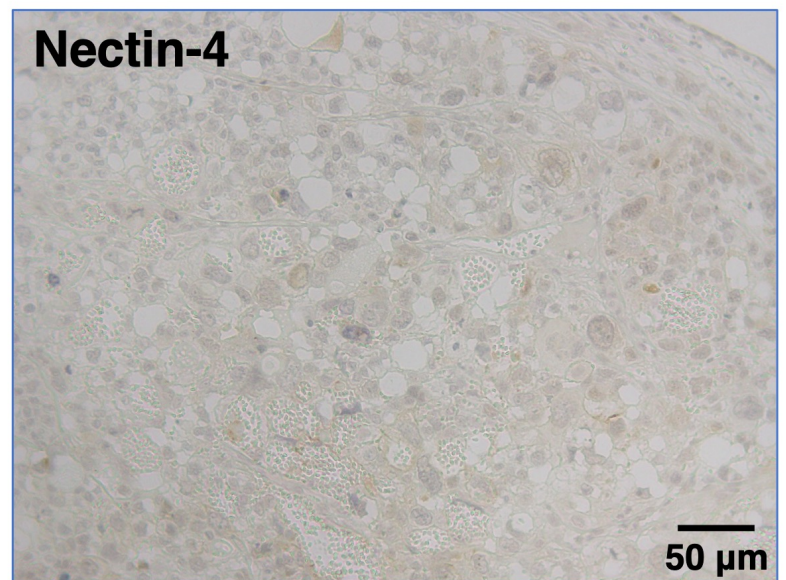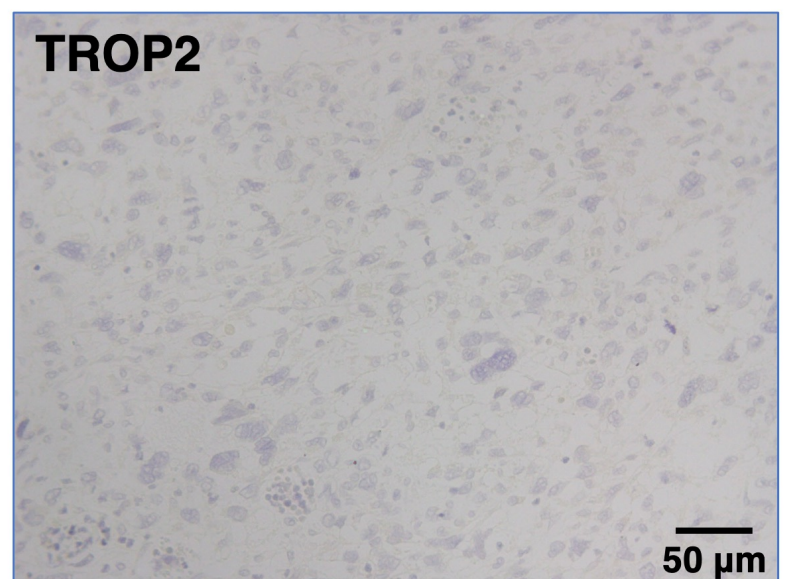

Supplement: Supplementary file 9 — Figure S7: Representative immunohistochemical staining patterns of markers associated with histological subtypes/divergent differentiation (S/DD) in upper tract urothelial carcinoma. Representative images of markers positively associated with S/DD are shown in the left panel, including PD‐L1, EGFR, and p53. Representative images of markers inversely associated with S/DD are shown in the right panel, including FGFR3, GATA3, Nectin‐4, and TROP2. Scale bars = 50 μm. [file IJU-33-0-s006.pdf]
