# Supplementary material for: Comprehensive Genomic Characterization Between Urothelial Carcinoma Subtypes/Divergent Differentiation (S/DD) and Pure Urothelial Carcinoma Using a Large‐Scale Japanese Genomic Panel Dataset
Source: Int J Urol. 2026 Jun 8;33(6):e70538. doi: 10.1111/iju.70538 (PMC13244187; doi:10.1111/iju.70538)

C-CAT mutation data

A Squamous differentiation (n=60)

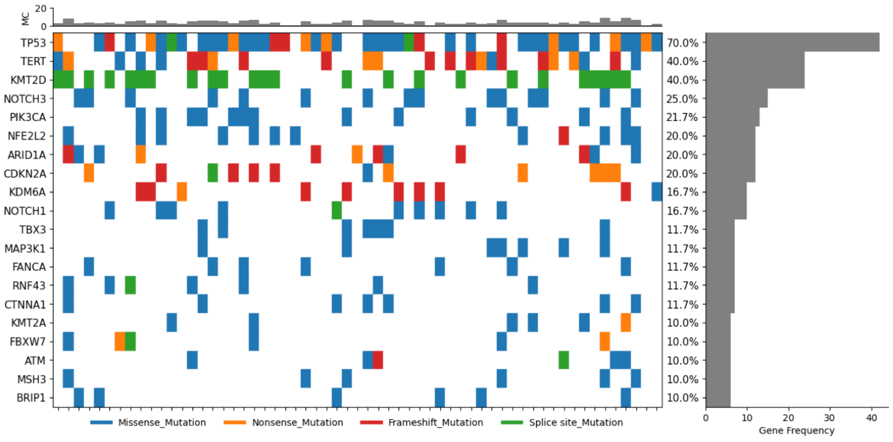

B Glandular differentiation (n=47)

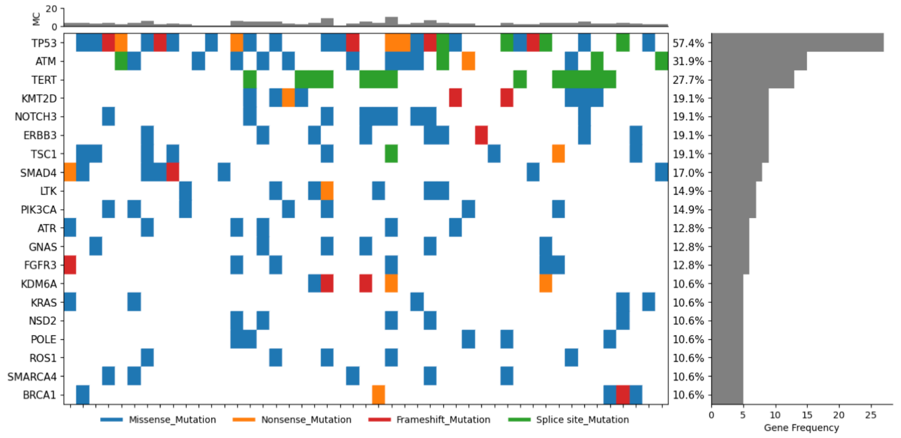

C Neuroendocrine carcinoma (n=71)

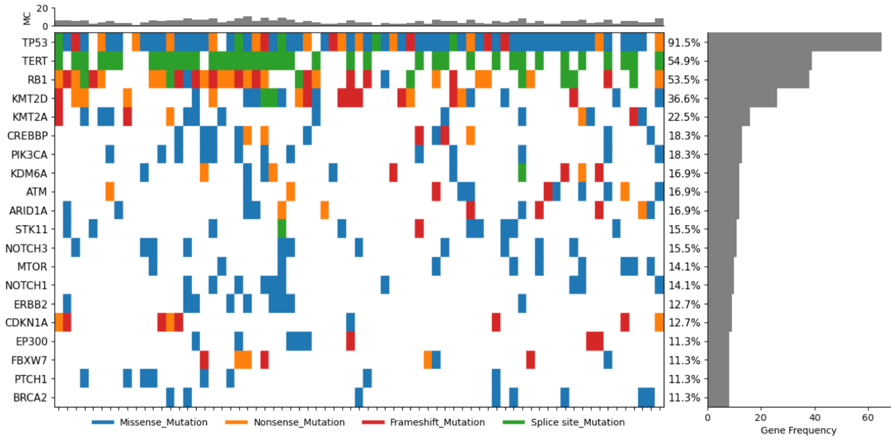

D Plasmacytoid/Signet Ring Cell (n=18)

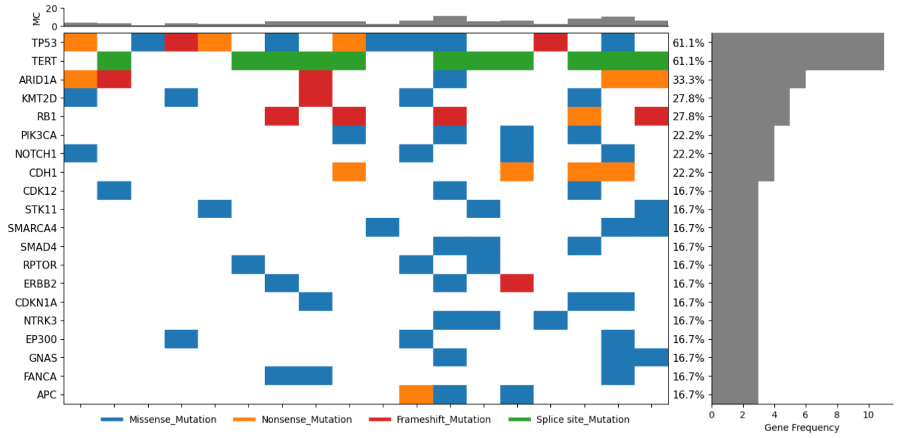

E Micropapillary (n=9)

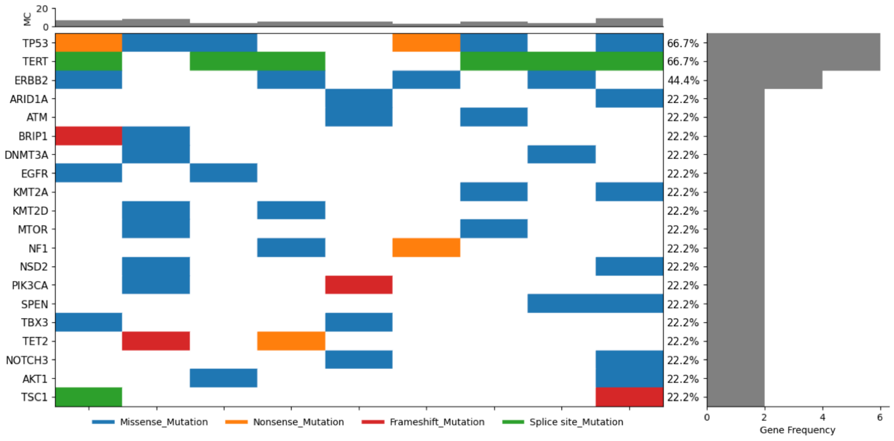

F Other histological subtype (n=14)

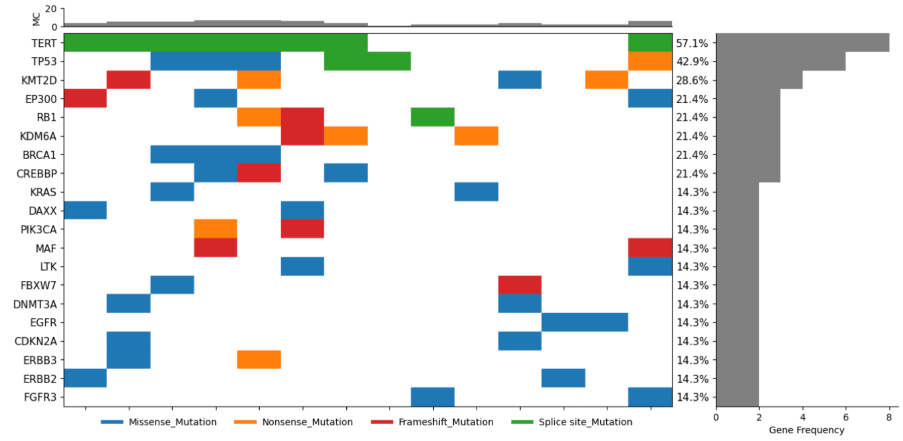

Supplement: Supplementary file 6 — Figure S4: Genomic profiles of urothelial carcinoma with subtypes/divergent differentiation (S/DD), stratified by each histological subtype, in the C‐CAT dataset. Oncoplots showing somatic mutations in frequently altered genes are presented for (A) squamous differentiation (n = 60), (B) glandular differentiation (n = 47), (C) neuroendocrine carcinoma (n = 71), (D) plasmacytoid/signet ring cell carcinoma (n = 18), (E) micropapillary carcinoma (n = 9), and (F) other histological subtypes (n = 14). [file IJU-33-0-s004.pdf]
