# Supplementary material for: Comprehensive Genomic Characterization Between Urothelial Carcinoma Subtypes/Divergent Differentiation (S/DD) and Pure Urothelial Carcinoma Using a Large‐Scale Japanese Genomic Panel Dataset
Source: Int J Urol. 2026 Jun 8;33(6):e70538. doi: 10.1111/iju.70538 (PMC13244187; doi:10.1111/iju.70538)
Supplement: Supplementary file 7 — Figure S5: Genomic profiles of urothelial carcinoma with subtypes/divergent differentiation (S/DD), stratified by each histological subtype, in the C‐CAT dataset. Oncoplots showing copy number alterations (CNAs) in frequently altered genes are presented for (A) squamous differentiation (n = 47), (B) glandular differentiation (n = 34), (C) neuroendocrine carcinoma (n = 53), (D) plasmacytoid/signet ring cell carcinoma (n = 12), (E) micropapillary carcinoma (n = 6), and (F) other histological subtypes (n = 10). [file IJU-33-0-s010.pdf]

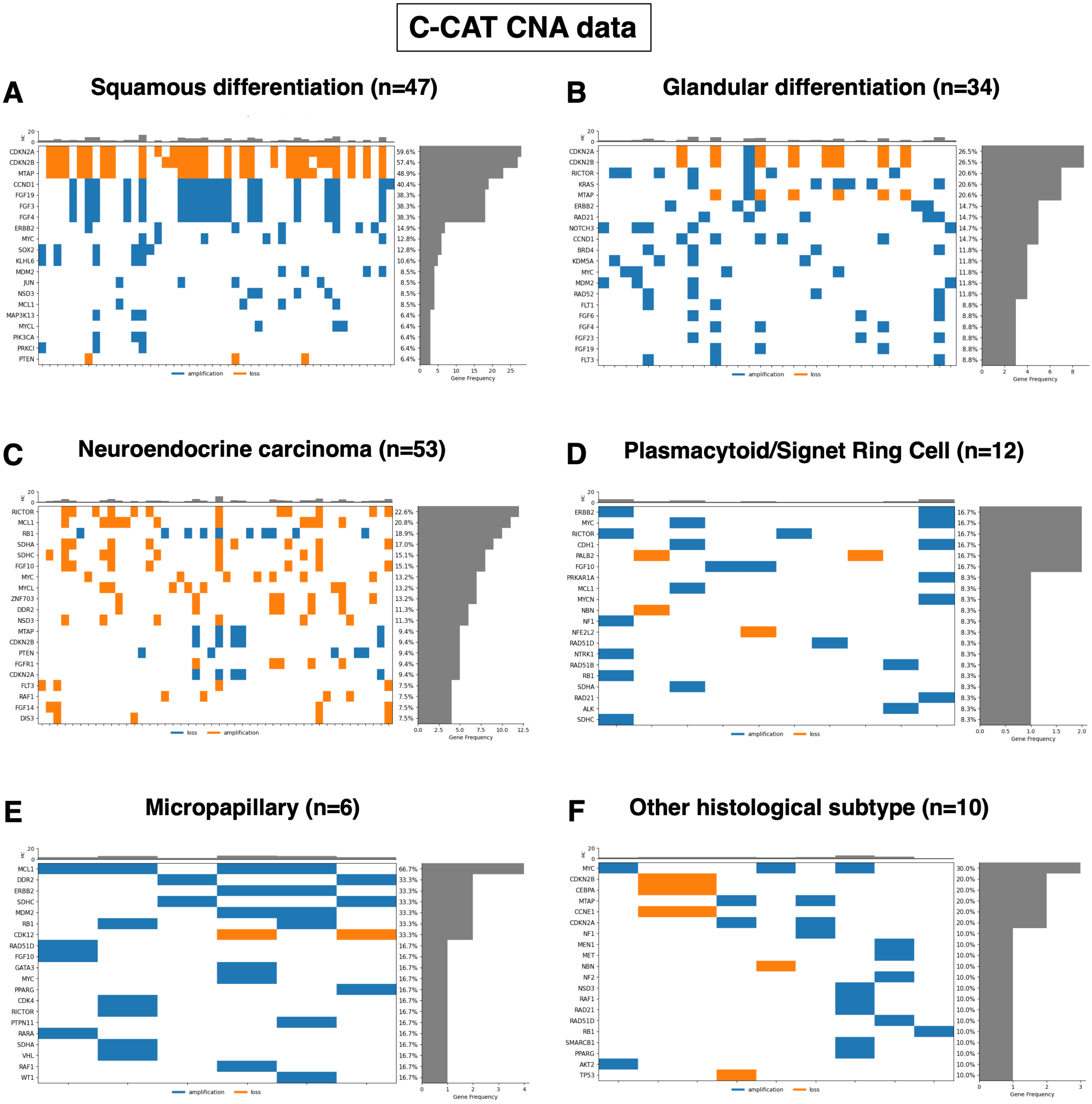
C-CAT CNA data
A
Squamous differentiation (n=47)
CDKN2A 59.6%
CDKN2B 57.4%
MTAP 48.9%
CCND1 40.4%
FGF19 38.3%
FGF3 38.3%
FGF4 38.3%
ERBB2 14.9%
MYC 12.8%
SOX2 12.8%
KLHL6 10.6%
MDM2 8.5%
JUN 8.5%
NSD3 8.5%
MCL1 8.5%
MAP3K13 6.4%
MYCL 6.4%
PIK3CA 6.4%
PRKCI 6.4%
PTEN 6.4%
amplification
loss
Gene Frequency
B
Glandular differentiation (n=34)
CDKN2A 26.5%
CDKN2B 26.5%
RICTOR 20.6%
KRAS 20.6%
MTAP 20.6%
ERBB2 14.7%
RAD21 14.7%
NOTCH3 14.7%
CCND1 14.7%
BRD4 11.8%
KDM5A 11.8%
MYC 11.8%
MDM2 11.8%
RAD52 11.8%
FLT1 8.8%
FGF6 8.8%
FGF4 8.8%
FGF23 8.8%
FGF19 8.8%
FLT3 8.8%
amplification
loss
Gene Frequency
C
Neuroendocrine carcinoma (n=53)
RICTOR 22.6%
MCL1 20.8%
RB1 18.9%
SDHA 17.0%
SDHC 15.1%
FGF10 15.1%
MYC 13.2%
MYCL 13.2%
ZNF703 13.2%
DDR2 11.3%
NSD3 11.3%
MTAP 9.4%
CDKN2B 9.4%
PTEN 9.4%
FGFR1 9.4%
CDKN2A 9.4%
FLT3 7.5%
RAF1 7.5%
FGF14 7.5%
DIS3 7.5%
loss
amplification
Gene Frequency
D
Plasmacytoid/Signet Ring Cell (n=12)
ERBB2 16.7%
MYC 16.7%
RICTOR 16.7%
CDH1 16.7%
PALB2 16.7%
FGF10 16.7%
PRKAR1A 8.3%
MCL1 8.3%
MYCN 8.3%
NBN 8.3%
NF1 8.3%
NFE2L2 8.3%
RAD51D 8.3%
NTRK1 8.3%
RAD51B 8.3%
RB1 8.3%
SDHA 8.3%
RAD21 8.3%
ALK 8.3%
SDHC 8.3%
amplification
loss
Gene Frequency
E
Micropapillary (n=6)
MCL1 66.7%
DDR2 33.3%
ERBB2 33.3%
SDHC 33.3%
MDM2 33.3%
RB1 33.3%
CDK12 33.3%
RAD51D 16.7%
FGF10 16.7%
GATA3 16.7%
MYC 16.7%
PPARG 16.7%
CDK4 16.7%
RICTOR 16.7%
PTPN11 16.7%
RARA 16.7%
SDHA 16.7%
VHL 16.7%
RAF1 16.7%
WT1 16.7%
amplification
loss
Gene Frequency
F
Other histological subtype (n=10)
MYC 30.0%
CDKN2B 20.0%
CEBPA 20.0%
MTAP 20.0%
CCNE1 20.0%
CDKN2A 20.0%
NF1 10.0%
MEN1 10.0%
MET 10.0%
NBN 10.0%
NF2 10.0%
NSD3 10.0%
RAF1 10.0%
RAD21 10.0%
RAD51D 10.0%
RB1 10.0%
SMARCB1 10.0%
PPARG 10.0%
AKT2 10.0%
TP53 10.0%
amplification
loss
Gene Frequency
