# Supplementary material for: Comprehensive Genomic Characterization Between Urothelial Carcinoma Subtypes/Divergent Differentiation (S/DD) and Pure Urothelial Carcinoma Using a Large‐Scale Japanese Genomic Panel Dataset
Source: Int J Urol. 2026 Jun 8;33(6):e70538. doi: 10.1111/iju.70538 (PMC13244187; doi:10.1111/iju.70538)
Supplement: Supplementary file 14 — Table S5: Comparison of each urothelial carcinoma subtypes/divergent differentiation (S/DD) and pure urothelial carcinoma. [file IJU-33-0-s007.docx]

Table S5 Comparison of each urothelial carcinoma subtypes/divergent differentiation (S/DD) and pure urothelial carcinoma

| Subtype | Key alterations |
| --- | --- |
| **Mutation data** |  |
| Squamous differentiation (n=60) | TP53↑, TERT↓ |
| Glandular differentiation (n=47) | ATM↑, TERT↓↓, KMT2D↓↓ |
| Neuroendocrine carcinoma (n=71) | TP53↑↑, RB1↑↑, KMT2A↑ |
| Plasmacytoid/Signet Ring Cell (n=18) | TERT↑, ARID1A↑, RB1↑, KMTD2↓ |
| Micropapillary (n=9) | TERT↑, ERBB2↑↑ |
| Other histological subtype (n=14) | TP53↓ |
| **CNA data** |  |
| Squamous differentiation (n=47) | CDKN2A↑, CDKN2B↑, MTAP↑, CCND1↑, FGF19↑, FGF4↑, FGF3↑ |
| Glandular differentiation (n=34) | RICTOR↑, KRAS↑, NOTCH3↑, CDKN2A↓, CDKN2B↓, MTAP↓ |
| Neuroendocrine carcinoma (n=53) | RICTOR↑, MCL1↑, RB1↑, CDKN2A↓↓, CDKN2B↓↓, MTAP↓↓ |
| Plasmacytoid/Signet Ring Cell (n=12) | MYC↑, CDH1↑, PALB2↑, CDKN2A↓↓, CDKN2B↓↓, MTAP↓↓ |
| Micropapillary (n=6) | MCL1↑↑, DDR2↑, ERBB2↑, SDHC↑, MDM2↑, RB1↑ |
| Other histological subtype (n=10) | MYC↑, CDKN2A↓↓, CDKN2B↓↓, MTAP↓ |

Arrows indicate approximate differences in alteration frequency relative to pure urothelial carcinoma (↑/↓ ≥10%; ↑↑/↓↓ ≥20%).
